# Pathobiont-driven antibody sialylation through IL-10 undermines vaccination

**DOI:** 10.1172/JCI179563

**Published:** 2024-12-16

**Authors:** Chih-Ming Tsai, Irshad A. Hajam, J.R. Caldera, Austin W.T. Chiang, Cesia Gonzalez, Du, Biswa Choudhruy, Haining Li, Emi Suzuki, Fatemeh Askarian, Ty’Tianna Clark, Brian Lin, Igor H. Wierzbicki, Angelica M. Riestra, Douglas J. Conrad, David J. Gonzalez, Victor Nizet, Nathan E. Lewis, George Y. Liu

**Affiliations:** 1 Division of Infectious Diseases, Department of Pediatrics, University of California, La Jolla, California, USA.; 2 Immunology Center of Georgia and Department of Medicine, Augusta University, Augusta, Georgia, USA.; 3 Glycobiology Research and Training Center, UCSD, La Jolla, California, USA.; 4 Department of Bioengineering, University of California, La Jolla, California, USA.; 5 Division of Gastroenterology, Department of Pediatrics, UCSD, La Jolla, California, USA.; 6 Division of Gastroenterology, Rady Children’s Hospital, San Diego, California, USA.; 7 Division of Host-Microbe Systems & Therapeutics, Department of Pediatrics, UC San Diego School of Medicine, La Jolla, California, USA.; 8 Department of Biology, San Diego State University, San Diego, California, USA.; 9 Skaggs School of Pharmacy and Pharmaceutical Sciences, UCSD, La Jolla, California, USA.; 10 Division of Pulmonary, Critical Care and Sleep Medicine, UCSD, La Jolla, California, USA.; 11 Division of Infectious Diseases, Rady Children’s Hospital, San Diego, California, USA.

**Keywords:** Immunology, Infectious disease, Adaptive immunity, Imprinting

## Abstract

The pathobiont *Staphylococcus aureus* (Sa) induces nonprotective antibody imprints that underlie ineffective staphylococcal vaccination. However, the mechanism by which Sa modifies antibody activity is not clear. Herein, we demonstrate that IL-10 is the decisive factor that abrogates antibody protection in mice. Sa-induced B10 cells drive antigen-specific vaccine suppression that affects both recalled and de novo developed B cells. Released IL-10 promotes STAT3 binding upstream of the gene encoding sialyltransferase ST3gal4 and increases its expression by B cells, leading to hyper-α2,3sialylation of antibodies and loss of protective activity. IL-10 enhances α2,3sialylation on cell-wall–associated IsdB, IsdA, and MntC antibodies along with suppression of the respective Sa vaccines. Consistent with mouse findings, human anti-Sa antibodies as well as anti-pseudomonal antibodies from cystic fibrosis subjects (high IL-10) are hypersialylated, compared with anti–*Streptococcus pyogenes* and pseudomonal antibodies from normal individuals. Overall, we demonstrate a pathobiont-centric mechanism that modulates antibody glycosylation through IL-10, leading to loss of staphylococcal vaccine efficacy.

## Abstract 2

There hasn’t been a successful staphylococcal vaccine to date. We show that S. aureus induces IL-10 which abrogates protective anti-staphylococcal antibody function through hyper-sialylation.

## Introduction

*Staphylococcus aureus* (Sa) is a leading agent of human bacterial skin, soft tissue, and invasive infections. The continual spread of antibiotic resistance, particularly in association with methicillin-resistant Sa (MRSA), has prompted a concerted effort to devise a viable human vaccine (1, 2). Despite the demonstrated efficacy of numerous anti-Sa vaccine candidates in naive mice, approximately 30 human clinical trials have failed to yield a functional vaccine (1, 2), for unclear reasons. Laboratory animals are predominantly naive to human Sa, which stands in stark contrast to humans who encounter Sa from early infancy onward (3). As a superbly adapted “pathobiont,” Sa has evolved intricate mechanisms facilitating its coexistence with the host (4–7).

Our prior work has revealed that Sa modification of its own surface peptidoglycan blunts the host’s development of anti-staphylococcal Th17 immunity, thereby permitting subsequent Sa infections to proceed without a reduction in pathogen burden (8). Consequently, we posit that anti-Sa vaccines developed in naive rodents may inadequately recapitulate trial outcomes in human who have prior exposure to Sa. In a recent investigation, we demonstrated that prior infections induced by i.p., i.v., or s.c. Sa administration impede the effectiveness of the subsequently administered IsdB vaccine (9). Our findings indicated that Sa infections generate nonprotective antibody imprints that, upon IsdB vaccination, lead to suboptimal vaccine responses (9). Furthermore, we have extended these observations to vaccines targeting 5 other Sa cell-wall–associated antigens (9, 10). Thus, imprinting emerges as a plausible explanation for the widespread failure of Sa vaccines.

While we had established that humoral imprinting interferes with effective vaccination, the mechanism by which the pathobiont Sa renders anti-Sa antibodies ineffectual across a spectrum of vaccines remained unclear. Our prior study found that IsdB antibodies exhibited nonprotective characteristics, primarily as they preferentially targeted the nonneutralizing NEAT1 domain of IsdB (9). Additionally, IsdB antibodies displayed elevated α2,3 sialylation, which impeded opsonophagocytosis (9).

Glycosylation of the IgG-Fc plays a pivotal role in regulating antibody function through Fc-mediated effector functions (11). Generally, IgGs lacking galactose (agalactosylated), fucose (afucosylated), or carrying bisecting GlcNAc modifications are associated with enhanced proinflammatory properties. In contrast, galactosylated and sialylated IgGs are correlated with attenuated inflammatory responses (12, 13). Moreover, sialylated IgG is linked to immunosuppressive effector functions (14–16). Modified IgG glycosylation is linked to autoimmunity and persistent infection and has a reported role in modulating the inflammatory cytokine profile (17). Some reports have demonstrated a direct role of cytokines in antibody glycosylation that leads to functional consequences. For example, IL-23 directs antibody glycosylation that promotes inflammation (18). Conversely, in CD8^+^ T cells, IL-10 enhances N-glycan branching to decrease antigen sensitivity in a cytomegalovirus infection model (19).

Given the broad effect of cytokines and their reported roles in the regulation of glycan expression, we investigate here the hypothesis that a cytokine induced by Sa regulates antibody glycosylation, thereby promoting a generalized inefficacy of Sa vaccines.

## Results

### Sa-induced IL-10 impacts IsdB antibody protective function.

**Figure 1:**
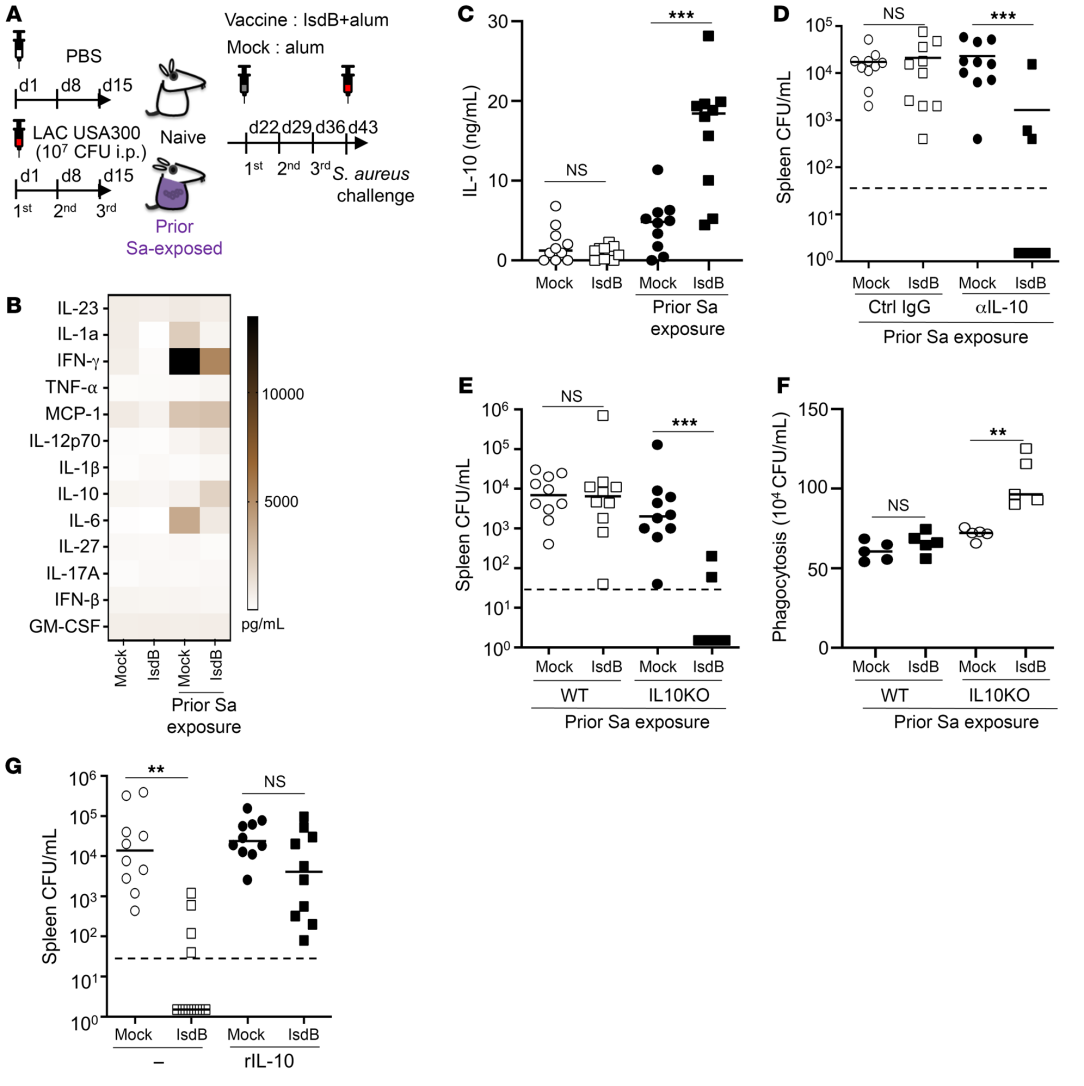
IL-10 plays a critical role in retuning protective IsdB antibody function. (**A**) Experimental setup. C57BL/6 mice were injected i.p. with Sa (LAC) or saline, immunized i.p., then challenged with LAC i.p. (**B**) Effect of IsdB vaccination on serum cytokines, measured by multiplex cytokine assay performed 1 day after Sa infection. IsdB, naive mice vaccinated with IsdB plus alum; mock, naive mice given alum; Sa/IsdB, LAC-infected mice vaccinated with IsdB/alum; Sa/mock, LAC-infected mice given alum alone. The heatmap graph represents mean values from *n* = 5 mice per group. (**C**) Serum IL-10 protein 24 hours after infection. Experiment performed as in Figure 1A (*n* = 10 per mouse group from 2 independent experiments). (**D**) IL-10–neutralizing antibody restores IsdB antibody protection. Mice were infected as in Figure 1A and vaccinated with IsdB with or without αIL-10 antibody. Seven days after, serum was adoptively transferred into naive mice followed by LAC challenge (*n* = 10 per mouse group from 2 independent experiments). (**E**) IsdB vaccination induced protective antibodies in IL-10^–/–^ mice. C57BL/6 mice or congenic IL-10^–/–^ mice were infected and vaccinated as in **A**. Serum was adoptively transferred 7 days after vaccination to assess for anti-Sa immunity by LAC challenge (*n* = 10 per mouse group from 3 independent experiments). (**F**) Opsonophagocytosis of Sa (LAC) by primary mouse neutrophils in the presence of immunized sera from **E**. Mean values ± SD from 3 independent experiments. (**G**) IL-10 limits IsdB vaccine efficacy. Naive mice were administered IL-10 or control with IsdB vaccination. Serum was assessed for anti-Sa immunity by adoptive transfer into naive mice followed by LAC challenge (*n* = 10 per mouse group from 2 independent experiments). Bars represent group median; dashed lines indicate the limit of detection (**D**, **E** and **G**). **P < 0.01; ***P < 0.001, 1-way ANOVA followed by Bonferroni’s multiple-comparison adjustment (**C**–**G**).

To investigate our hypothesis linking a Sa-induced cytokine response to antibody glycosylation, we initially examined the cytokine milieu associated with effective and noneffective vaccine antibody responses in naive and Sa-exposed mice, respectively. For this analysis, we used the well-characterized IsdB vaccination model established in our previous study (9), inspired by the real-world experience of a promising IsdB vaccine candidate that later proved to be unsuccessful in a major randomized placebo-controlled human trial (20). We administered 1 × 10^7^ CFUs of Sa (USA300 MRSA LAC strain) by i.p. injection to C57BL/6 mice once weekly for 3 weeks to establish robust humoral imprints. This was followed by 3 IsdB vaccinations in alum or alum alone, culminating in a final challenge with MRSA 7–10 days after the last vaccination (Figure 1A). Notably, IsdB-vaccinated mice exhibited an altered serum cytokine profile, regardless of the final systemic challenge, although the cytokine levels measured were significantly higher with the final Sa challenge (Figure 1B and Supplemental Figure 1A; supplemental material available online with this article; https://doi.org/10.1172/JCI179563DS1). Exposure to Sa alone increased serum IL-1α, IFN-γ, and IL-6. With IsdB vaccination, the mice exhibited reduced levels of these inflammatory cytokines, except for IL-10, which was increased.

The heightened IL-10 levels with Sa exposure/vaccination are particularly intriguing due to the strong association of IL-10 with Sa colonization, biofilm formation, reinfection, and mortality in bacteremia (8, 21–23). Confirming our findings, IsdB vaccination of Sa-exposed hosts, followed by a high dose of Sa, led to elevated serum IL-10 levels in a murine model of severe bacteremia (Figure 1C and Supplemental Figure 1B). To interrogate the role of IL-10 in vaccine suppression, we conducted the vaccination experiment in the presence or absence of IL-10–neutralizing antibody, followed by adoptive transfer of the serum into naive mice and subsequent Sa challenge to measure anti-Sa immunity associated with serum antibodies. IL-10–neutralizing antibody treatment had no effect on the titer of IsdB-specific antibodies (Supplemental Figure 1C) but restored the protective function of the transferred serum (Figure 1D and Supplemental Figure 1D). In a parallel IsdB vaccination experiment in WT and IL-10^–/–^ mice, we observed that genetic ablation of IL-10 resulted in the restoration of protective serum antibody function (Figure 1E and Supplemental Figure 1E). In vitro, sera from IL-10^–/–^ mice supported enhanced killing of Sa compared with sera from WT mice in an opsonophagocytosis assay (Figure 1F). Conversely, i.p. administration of recombinant IL-10 with IsdB vaccination in naive mice abrogated anti-Sa immunity conferred by the sera upon adoptive transfer into naive mice (Figure 1G and Supplemental Figure 1F). We conclude that IL-10 plays a critical role in regulating the protective function of antibodies.

### B suppressor cell phenotype and impact on de novo anti-Sa B cell development and function.

**Figure 2:**
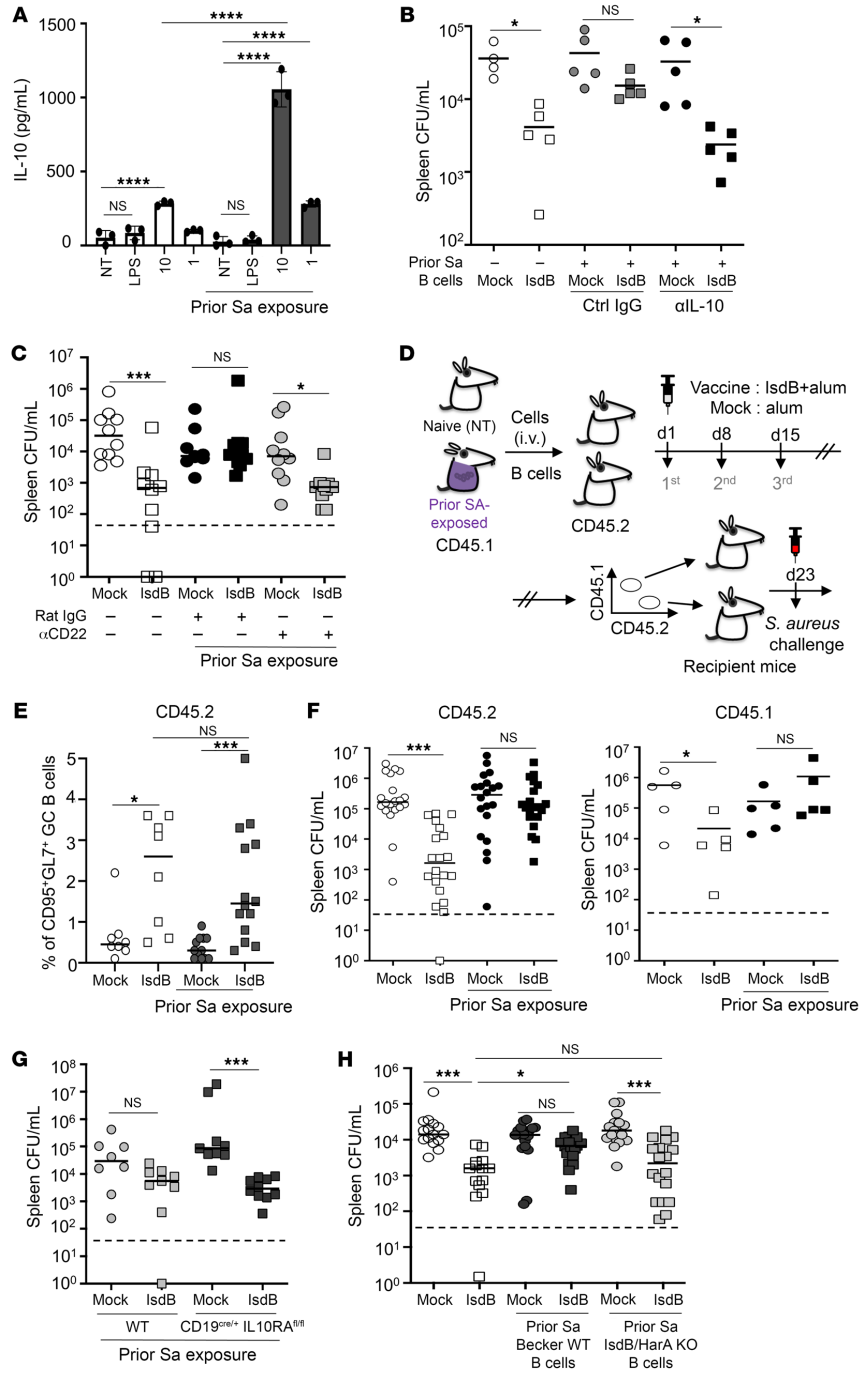
B10 cells abrogate humoral protection induced by IsdB vaccination. (**A**) IL-10 in culture supernatant 16 hours after stimulation of naive or Sa-experienced splenic B cells with LPS or heat-killed Sa at indicated MOI.NT, nontreated. (**B**) αIL-10 antibody abrogates suppressive B cell effect on IsdB vaccination. Naive mice administered B cells from Sa-exposed mice were IsdB vaccinated with/without αIL-10 antibody, then challenged as per Figure 1A (*n* = 4–5 per mouse group). (**C**) αCD22 antibody depletion of B10 cells a day prior to immunizations restores IsdB vaccine efficacy. (*n* = 7–10 per mouse group from 3 independent experiments). (**D**) Experimental setup for **E** and **F**. Splenic B cells were transferred from naive/Sa-infected CD45.1 mice into naive CD45.2 mice. Recipients were IsdB immunized, and splenic B cells were analyzed after 7 days (**E**). CD45.1 or CD45.2 B cells were then transferred into naive C57BL/6 mice followed by Sa challenge (**F**). (**E**) No difference in percentage (**F**) but lack of protective function of de novo developed (CD45.2) splenic B cells in mice exposed to suppressive B cells. (**E** and **F**) CD45.1, *n* = 5 per group, CD45.2, *n* = 20 per group from 4 independent experiments. (**G**) Bacterial burden in WT CD19^cre/+^ mice or CD19^cre/+^ IL-10RA^fl/fl^ that were infected and IsdB vaccinated as per Figure 1A (*n* = 8–10 per mouse group from 3 independent experiments). (**H**) B cells do not suppress IsdB vaccination when IsdB/HarA mutant Becker strain is used in prior infection. Infection/vaccination as per Figure 1A followed by antibody transfer. WT Becker was used in final challenge. (*n* = 15 per mouse group from 3 independent experiments). Bars represent group means; error bars represent means ± SD (**A**). Dashed lines indicate the limit of detection (**B** to **C** and **E** to **H**). *P < 0.05; ***P < 0.001; ****P < 0.0001, 1-way ANOVA followed by Bonferroni’s multiple-comparison adjustment (**A** to **C** and **E** to **H**).

In a prior study, we demonstrated that adoptive transfer of B cells from Sa-infected mice was sufficient to nullify the protective effect of IsdB vaccination in naive recipient mice (9). To query the link between B cell–mediated suppression of IsdB vaccination and IL-10, we exposed splenic B cells from naive and Sa-exposed mice to ex vivo heat-killed Sa challenge (Figure 2A). Compared with naive splenic B cells, B cells from Sa-exposed mice released 3- to 4-fold higher levels of IL-10 when stimulated with heat-killed Sa. In vivo, the adoptive transfer of B cells from Sa-exposed mice induced vaccine suppression as observed previously, but coadministration of anti–IL-10 antibody with vaccination in the recipient restored vaccine protection against Sa challenge (Figure 2B and Supplemental Figure 2A).

B10 cells are a subset of human or mouse B cells that regulate immune functions through the secretion of IL-10. To further confirm that B10 cells are responsible for vaccine suppression, we utilized anti-CD22 antibody to deplete B10 cells (24) from mice previously exposed to Sa. Subsequent to anti-CD22 antibody administration, vaccination in these mice regained effectiveness (Figure 2C and Supplemental Figure 2B). In a separate study addressing the involvement of T cells in vaccine interference (our unpublished observations), the depletion of IL-10–secreting T cells similarly restored the efficacy of the IsdB vaccine, suggesting IL-10 as the common source of vaccine suppression.

While our findings indicate that IL-10 released by B cell imprints undermines IsdB vaccination and is responsible for the generation of nonprotective antibodies, it remains unclear how B10 cells affect the quantitative or qualitative aspects of de novo B cell development. To address this, we performed an adoptive transfer of B cells from Sa-exposed CD45.1 allotype-marked mice into CD45.2 congenic mice, followed by IsdB vaccination of the cell recipients. Strikingly, there was no reduction in the percentage of de novo developed B cells (CD45.2) in these recipient mice compared with mouse-transferred CD45.1 naive B cells. To determine if there were functional changes in the de novo developed B cells, 7 days after the final vaccination, CD45.1 and CD45.2 splenic B cells were transferred into separate naive recipient mice and evaluated for anti-Sa immunity against a Sa challenge (Figure 2D). In contrast to the lack of quantitative differences, de novo–developed B cells exhibited a loss of protective function (Figure 2, E and F, and Supplemental Figure 2, C and D).

To unravel how B cells lose protective function in the context of IL-10, we obtained B cell–specific IL-10RA knockout mice (CD19^cre/+^ IL-10RA^fl/fl^ mice) by crossing CD19 cre mice with IL-10RA flox mice (25) and evaluated IsdB vaccine interference in CD19^cre/+^ WT and CD19^cre/+^ IL-10RA^fl/fl^ mice. The IL-10RA–KO mice displayed no vaccine interference compared with CD19^cre/+^ WT mice (Figure 2G and Supplemental Figure 2E). Furthermore, we established that humoral vaccine suppression was antigen specific, as B cells isolated from mice infected thrice with the WT or isogenic IsdB and homologue double mutant (IsdB/HarA mutant) (26) failed to suppress IsdB vaccination upon adoptive transfer, in contrast with B cells isolated from mice infected thrice with the WT isogenic Sa strain (Figure 2H and Supplemental Figure 2F).

To identify the B cell subtype responsible for conferring suppression, we measured the frequency and number of total B cells (B220^+^), follicular (FO) B cells (CD19^+^CD2^low^CD23^+^), marginal zone (MZ) B cells, (CD19^+^CD21^high^CD23^–^), and B10 cells (CD19^+^CD1d^high^CD5^+^) in naive or Sa-exposed mice (27, 28). Notably, there was a significant increase in cell numbers and the population of MZ B cells and B10 cells, but not total B cells and FO B cells, in Sa-exposed mice compared with naive mice (Supplemental Figure 3, A and B). Consistent with these findings, both B10 cells and MZ B cells released robust IL-10 in response to heat-killed Sa compared with FO B cells (Supplemental Figure 3, C and D). Furthermore, adoptive transfer of total B cells or B10 cells, but not FO B cells, from Sa-exposed mice, abrogated the protective effect of IsdB vaccination in naive recipient mice, thereby corroborating the major suppressive roles of B10 cells (Supplemental Figure 4, A and B). Adoptive transfer of MZ B cells blunted IsdB vaccine protection in the spleen but not the kidneys. We speculate that MZ B cells preferentially home to the spleen and therefore exert their suppressive effect primarily in the spleen but not the kidneys.

### IL-10 selectively promotes α2,3 sialylation of IsdB antibody.

Having established the decisive role of IL-10 in reshaping the nonprotective function of IsdB antibodies and demonstrated the criticality of IL-10R expressed on B cells for suppression (Figure 2G and Supplemental Figure 2E), we proceeded to investigate if and how IL-10 and its signaling remodel IsdB antibodies for inactivity.

Previously, we demonstrated that the IsdB Fab targets the nonneutralizing NEAT1 domain of IsdB, which is preferentially recalled with vaccination (9). Through single-cell analysis of antibody complementary determining region (CDR3), neutralizing IL-10 treatment is shown to have no significant effect on clonal imprinting in Sa-exposed IsdB-vaccinated mice, although there are few notable novel clones of unclear significance with anti–IL-10 antibody treatment (Supplemental Figure 5A).

In our previous study, we identified increased α2,3 sialylation on IsdB antibodies as a feature that reduces antibody opsonophagocytic killing (OPK) function (9). Although the effect of IL-10 on antibody glycosylation is unclear, IL-10 has been shown to desensitize CD8^+^ T cells by enhancing N-glycan branching on T cells (19). To investigate whether IL-10 modifies α2,3 sialylation, N glycan, or other glycans on IsdB antibodies, we purified IsdB-specific antibodies from mice that were naive or Sa exposed, then vaccinated with IsdB in the presence or absence of IL-10–neutralizing antibody. The glycosylation modifications on the antibodies were determined using lectin binding assays, notably, sambucus nigra lectin (SNA) for α-2,6 *N*-acetylneuraminic acid–galactose (Neu5Aca2-6 galactose) binding, maackia amurensis lectin II (MAA) for α-2,3 *N*-acetylneuraminic acid–galactose (Neu5Aca2-3 galactose) binding, or galactosyl (β-1,3) *N*-acetylgalactosamine binding, phaseolus vulgaris leucoagglutinin (PHA-L) for Mgat5-modified N-glycans binding, or erythrina cristagalli lectin (ECL) for *N*-acetyllactosamine binding.

**Figure 3:**
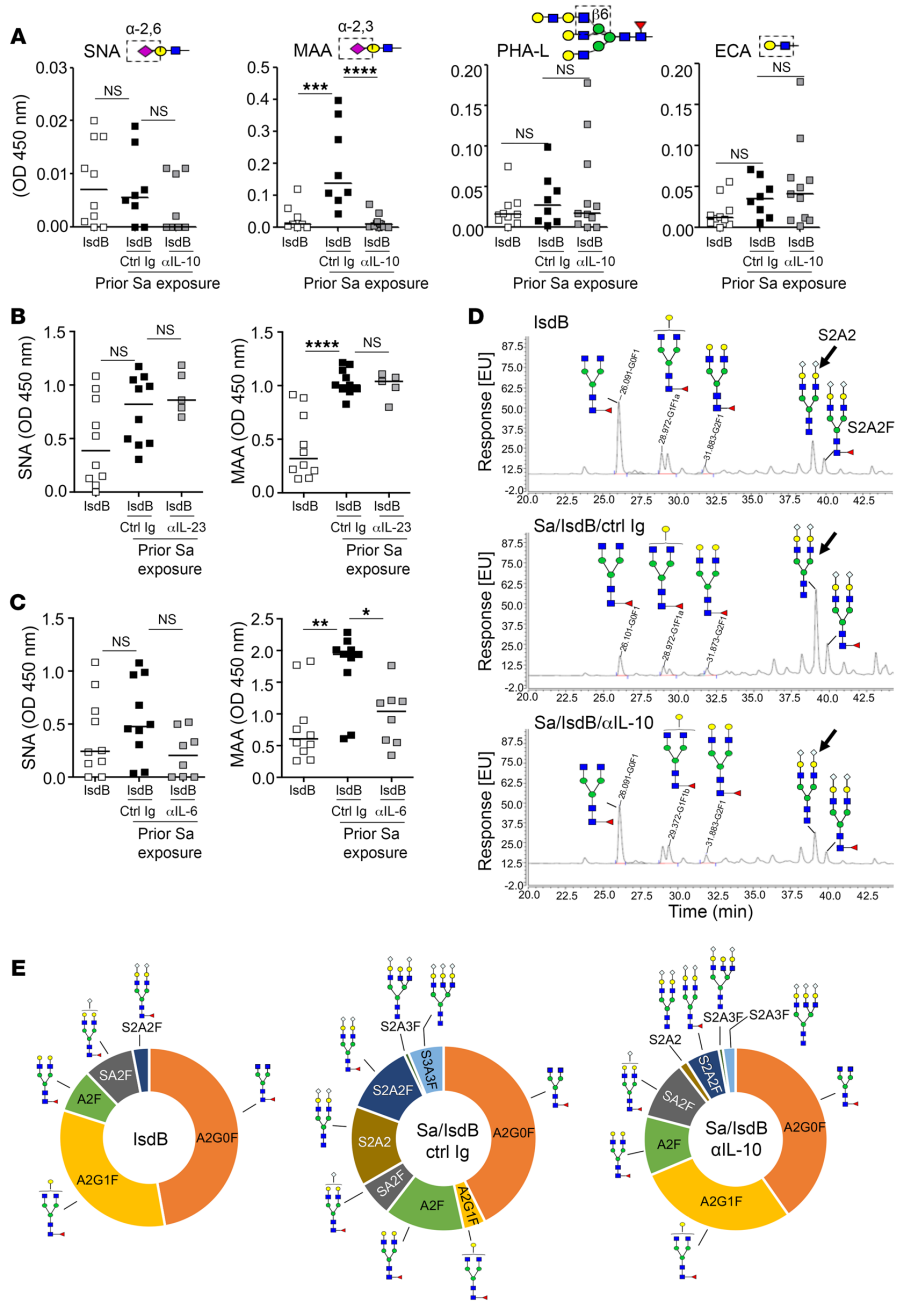
IL-10 modifies α2,3 sialylation on IsdB antibodies. (**A**) Effect of αIL-10 antibody treatment on IsdB antibody glycosylation. IsdB antibodies purified from mice in Figure 1D (treated with Ctrl IgG or αIL-10) and assessed by lectin ELISA to determine antibody glycan content. (**B** and **C**) Effect of IL-23– or IL-6–neutralizing antibody on IsdB antibody sialylation. Serum IsdB antibodies from IL-6 (**B**) or IL-23 (**C**) antibody-treated, Sa/IsdB vaccinated mice, per Figure 1D, were assessed for α-2,6 or α-2,3 sialylation by SNA and MAA lectin ELISA. (**D**) UPLC-FL analysis of N-glycans released from PNGaseF treatment of purified IsdB antibodies from mice in Figure 1D (treated with Ctrl IgG or αIL-10). (**E**) Pie charts showing percentage of N-glycans in Supplemental Figure 5F Glycan schematics used here and in all other figures follow the recommended symbol nomenclature for glycans (SNFG). Glycan nomenclature: blue, N-acetylglucosamine (GlcNAc); yellow, galactose (Gal); green, mannose (Man); pale blue, sialic acid (Neu5Gc or Neu5Ac); red, fucose (Fuc); A, antennae; S, sialic acid; F, fucose; G, galactose. Bars represent group median; each point represents an individual mouse (**A**–**C**). *P < 0.05; **P < 0.01; ***P < 0.001; ****P < 0.0001, 1-way ANOVA followed by Bonferroni’s multiple-comparison adjustment (**A**–**C**).

MAA binding (α2,3 sialylation) was notably elevated with vaccination of Sa-exposed mice, as reported previously (9), and was restored to protective IsdB antibody levels with anti–IL-10 treatment. We observed no significant modification of SNA, PHA-L, or erythrina cristagalli lectin (ECA) binding with anti–IL-10 treatment, suggesting that IL-10 has a selective role in the modulation of antibody α2,3 sialylation (Figure 3A).

Prior studies have demonstrated a regulatory role of the IL-23/TH17 axis on autoantibody sialylation in autoimmunity (18). Our cytokine profiling noted differences in IL-12, IL-23, and, most strikingly, IL-6 between control and IsdB vaccination in mice previously exposed to Sa. Therefore, we evaluated whether anti–IL-6, anti–IL-12, or anti–IL-23 antibodies can modify the level of sialylation on IsdB antibodies. Overall, we did not observe an effect of IL-12 or IL-23 on IsdB antibody sialylation (Supplemental Figure 5B and Figure 3B). By contrast, IL-6, like IL-10, enhanced the level of antibody α2,3 sialylation (Figure 3C). Blocking of IL-6, IL-12, or IL-23 in vivo did not alter IsdB vaccine efficacy (Supplemental Figure 5, C and D). Therefore, we conclude that these cytokines do not appreciably impact humoral vaccine responses.

We corroborated the glycan structures of IsdB antibodies using ultra-performance liquid chromatography with fluorescence (UPLC-FL), revealing that IsdB antibodies from Sa-infected/IsdB-vaccinated mice carried higher levels of sialylated glycans, S2A2 and S2A2F, compared with IsdB antibodies generated from IsdB-vaccinated mice, a condition that is restored with anti–IL-10 antibody treatment (Figure 3D and Supplemental Figure 5E). Likewise, analysis of N-glycan profiling via matrix-assisted laser desorption/ionization time-of-flight/time-of flight (MALDI-TOF/TOF) mass spectrometry demonstrated that IsdB antibodies from Sa/IsdB mice had elevated levels of sialylated glycans S2A2, S2A2F, S2A3F and S2A3F, compared with IsdB antibodies from naive/IsdB mice. Anti–IL-10 antibody treatment reduced sialylated glycans on IsdB antibodies (Figure 3E and Supplemental Figure 5F) and across most IgG subclasses in SA-exposed IsdB-vaccinated mice (Supplemental Figure 6).

### IL-10 promotes STAT3 binding upstream of St3gal and enhances its expression.

ST3gal mediates α2,3 sialylation, and ST6gal regulates α2,6 sialylation (29). Since IL-10R expressed on B cells is essential for humoral vaccine suppression, we next assessed whether IL-10 modified B cell sialyltransferase gene expression.

**Figure 4:**
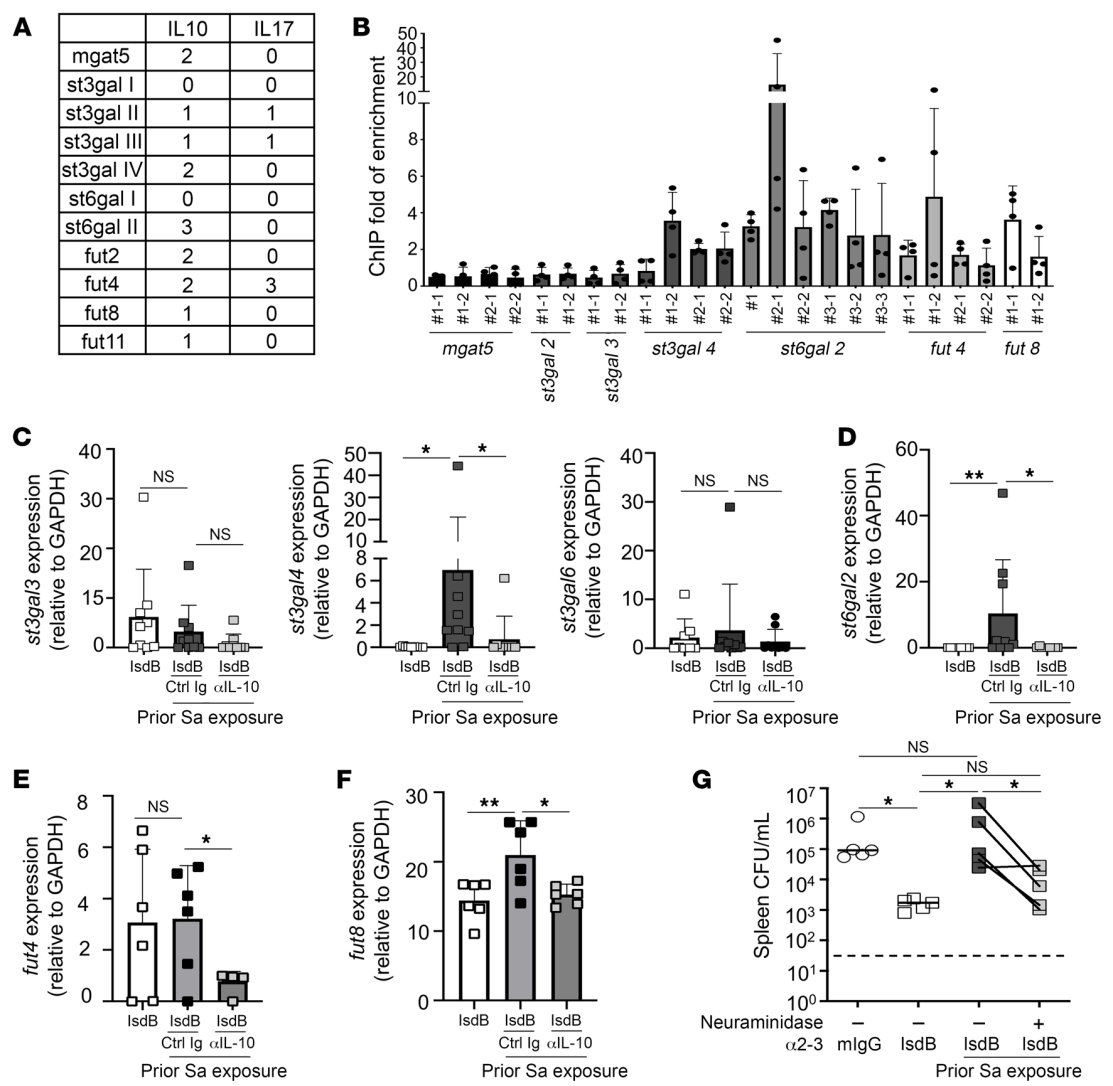
IL-10 promotes STAT3 binding to *St3gal4* promoter, which drives suppressive hypersialylation of IsdB antibodies. (**A**) Number of putative STAT3 (IL-10) and NF-κB (IL-17A) binding sites on glycotransferase genes. (**B**) Effect of recombinant IL-10 on DNA-binding activity of STAT3 in naive splenic B cells, assessed by ChIP quantitative PCR (ChIP-qPCR) analysis. (**C**–**F**) Effect of αIL-10 antibody treatment on splenic B cell *St3Gal* (**C**), *St6Gal* (**D**), or *Fut* (**E** and **F**) expression. Experiment performed as in Figure 1D. (**G**) Effect of IsdB antibody desialylation on anti-Sa immunity in vivo. Naive mice were injected with α2-3 neuraminidase- or control- treated, purified Sa/IsdB antibody, then infected with LAC (*n* = 5 per mouse group). Bars represent group means; each point represents an individual mouse; error bars represent means ± SD (**C**–**F**). Bar represents group means; error bars represent means ± SD (**B**). Bar represents group median; each point represents an individual mouse; dashed lines indicate the limit of detection (**G**). *P < 0.05; **P < 0.01, 1-way ANOVA followed by Bonferroni’s multiple-comparison adjustment (**C**–**G**).

STAT3 is a well-characterized transcription factor downstream of IL-10 (30). To determine whether STAT3 regulates glycotransferase expression, we searched for STAT3-binding sites (31), TTCNNNGAA, in the promoter region of glycotransferases (32). We identified putative STAT3-binding sites upstream of *Mgat5*, *St3gal2-4*, *St6al2*, and *Fut* genes. In comparison, the proinflammatory cytokine IL-17A, which is linked to sialyltransferase gene expression through NF-κB, had fewer putative NF-κB binding sites (the transcription factor downstream of IL-17A), GGGRNYYYCC, on *St3gal2-3* and *Fut4* (Figure 4A).

To verify STAT3 binds upstream of glycotransferase genes following IL-10 treatment, we utilized a ChIP assay to pull down STAT3-bound chromatin in naive B cells following IL-10 treatment or buffer control. We then used specific primers (Supplemental Figure 7A) to amplify STAT3-bound DNA fragments. Importantly, we found strong enrichment of *St3gal4* and *St6gal2* (Figure 4B), suggesting that IL-10/STAT3 could regulate sialylation in B cells. *Fut4* and *Fut8* were also enriched with IL-10 treatment.

To directly determine whether sialyltransferase genes are directly modulated by IL-10, we isolated splenic B cells from mice IsdB immunized as before in the presence or absence of anti–IL-10 antibodies, then measured the expression of *St3gal* (Figure 4C and Supplemental Figure 7B) and *St6gal* (Figure 4D and Supplemental Figure 7C). *St3gal4* expression increased with IsdB vaccination in Sa-exposed mice but not in naive mice. Furthermore, IL-10–neutralizing antibody treatment reduced *St3gal4*, pinpointing IL-10 as an important factor controlling its expression.

Relatedly, IL-10 regulates CD8^+^ T cell activity through *Mgat5* and TCR N-glycosylation in a model of lymphocytic choriomeningitis virus (LCMV) infection (19). Fc galactosylation and fucosylation, which are mediated by fucosyl (*fut*) and galactosyl (*b4galt*) transferases, also impact IgG-Fc functions (11, 33). Thus, we measured the effect of IL-10 on expression of these additional glycosyl transferases. We showed that prior Sa infection significantly affected B cell expression of *fut8*, *fut11*, and *b4galt1* (Figure 4F and Supplemental Figure 7, F and G), but not *Mgat5*, *fut4*, and *fut9* (Figure 4E and Supplemental Figure 7, D and E) after IsdB vaccination. IL-10–neutralizing antibody treatment reduced expression of fucosyl and terminal galactosyl transferases, thus implicating these glycans as additional modifiers of antibody function that are under the control of IL-10.

Finally, we interrogated the role of α2,3 sialylation on IsdB antibody function in vivo. With α2,3 neuraminidase treatment, we have previously restored OPK of Sa in vitro. Here, we verified that α2,3 neuraminidase treatment did not affect the affinity of IsdB antibodies generated in Sa/IsdB-vaccinated mice (Supplemental Figure 7H). In a model of i.p. Sa infection, we showed that the removal of α2,3 sialic acid on IsdB antibodies improved the clearance of Sa (Figure 4G and Supplemental Figure 7I).

### IL-10 promotes sialylation of anti-Sa antibodies and broad vaccine failure.

**Figure 5:**
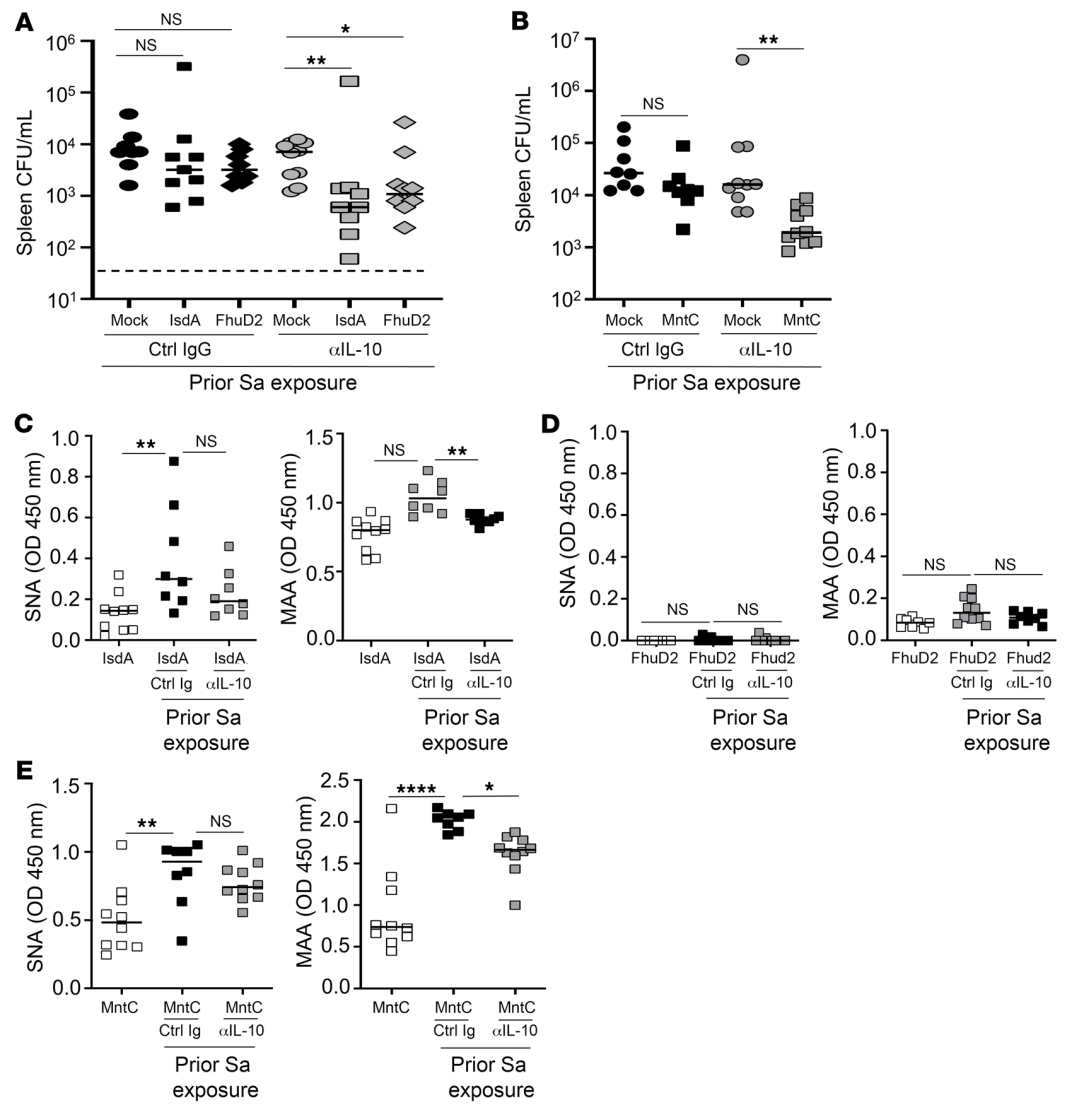
IL-10 promotes sialylation of anti-Sa antibodies and reduces anti-Sa vaccine efficacy. (**A** and **B**) Effect of αIL-10 antibody treatment on anti-Sa immunity conferred by IsdA, FhuD2, and MntC vaccination in Sa-exposed mice, performed as in Figure 1D (*n* = 7–10 per mouse group from 2 independent experiments). (**C**–**E**) Effect of αIL-10 antibody treatment on serum antibody sialylation after IsdA (**C**), FhuD2 (**D**), or MntC (**E**) vaccination in Sa-exposed mice, performed as in **A** and **B**, assessed by MAA and SNA lectin binding. Bars represent group median; each point represents an individual mouse; dashed lines indicate the limit of detection (**A** and **B**). Bar represents group median; each point represents an individual mouse (**C**–**E**). *P < 0.05; **P < 0.01; ****P < 0.0001, 1-way ANOVA followed by Bonferroni’s multiple-comparison adjustment (**A**–**E**).

We speculate that the nonspecific suppressive activity of IL-10 could represent a pathogen mechanism for broadly altering the effector functions of antibodies. Hence, we tested 3 additional Sa vaccines targeting cell wall–associated antigens IsdA, FhuD2, and MntC, each protective in naive mice but nonprotective in Sa-exposed mice (10). Corroborating prior data, all 3 vaccines were efficacious in naive mice but ineffective in Sa-exposed mice. Administration of IL-10–neutralizing antibodies at the time of vaccination restored efficacy of all 3 vaccines, although only in the spleen and not in the kidney for the FhuD2 vaccine (Figure 5, A and B, and Supplemental Figure 8, A and B).

We tested to determine whether IL-10–neutralizing antibody treatment also reversed specific antibody hypersialylation. As shown, anti–IL-10 reduced α2,3 but not α2,6 sialylation on IsdA and MntC antibodies (Figure 5, C and E). FhuD2 antibodies from Sa-exposed FhuD2-immunized mice showed a modest level of α2,3 sialylation that was not affected with IL-10 antibodies (Figure 5D).

It is notable that our study used i.p. as the primary immunization route, and therefore does not model the more commonly used i.m. route of immunization used in humans. We thus interrogated whether i.m. immunization with IsdB induces findings similar to those with i.p. immunization in naive and Sa-exposed mice (Supplemental Figure 9). Consistent with our prior findings, i.m. vaccination with IsdB was protective in naive but not in Sa-exposed mice (Supplemental Figure 9A); i.m. IsdB vaccination of Sa-exposed mice, similarly, induced higher levels of α2,3 sialylated antibodies and was nonprotective in OPK assay, compared with i.m vaccination of naive mice (Supplemental Figure 9, B and C). Furthermore, i.m. immunization appeared less effective and induced higher levels of IsdB antibody α2,3 sialylation compared with i.p. vaccination. Thus, IsdB vaccinations administered by both i.p. and i.m. routes are similarly impacted by prior Sa exposure, although the immunization route appears to drive differences in efficacy and antibody sialylation.

### Human anti-Sa antibody are hypersialylated.

Ultimately, mouse studies aim to elucidate the mechanisms of vaccine failures in humans. In mice, we demonstrate that antibody hypersialylation correlates with a lack of humoral protection. We thus examined whether hypersialylation is also a feature of human anti-Sa antibodies. In our prior studies (9, 10), we found that human serum IsdB, IsdA, and ClfA antibodies were ineffective in OPK of Sa by neutrophils.

**Figure 6:**
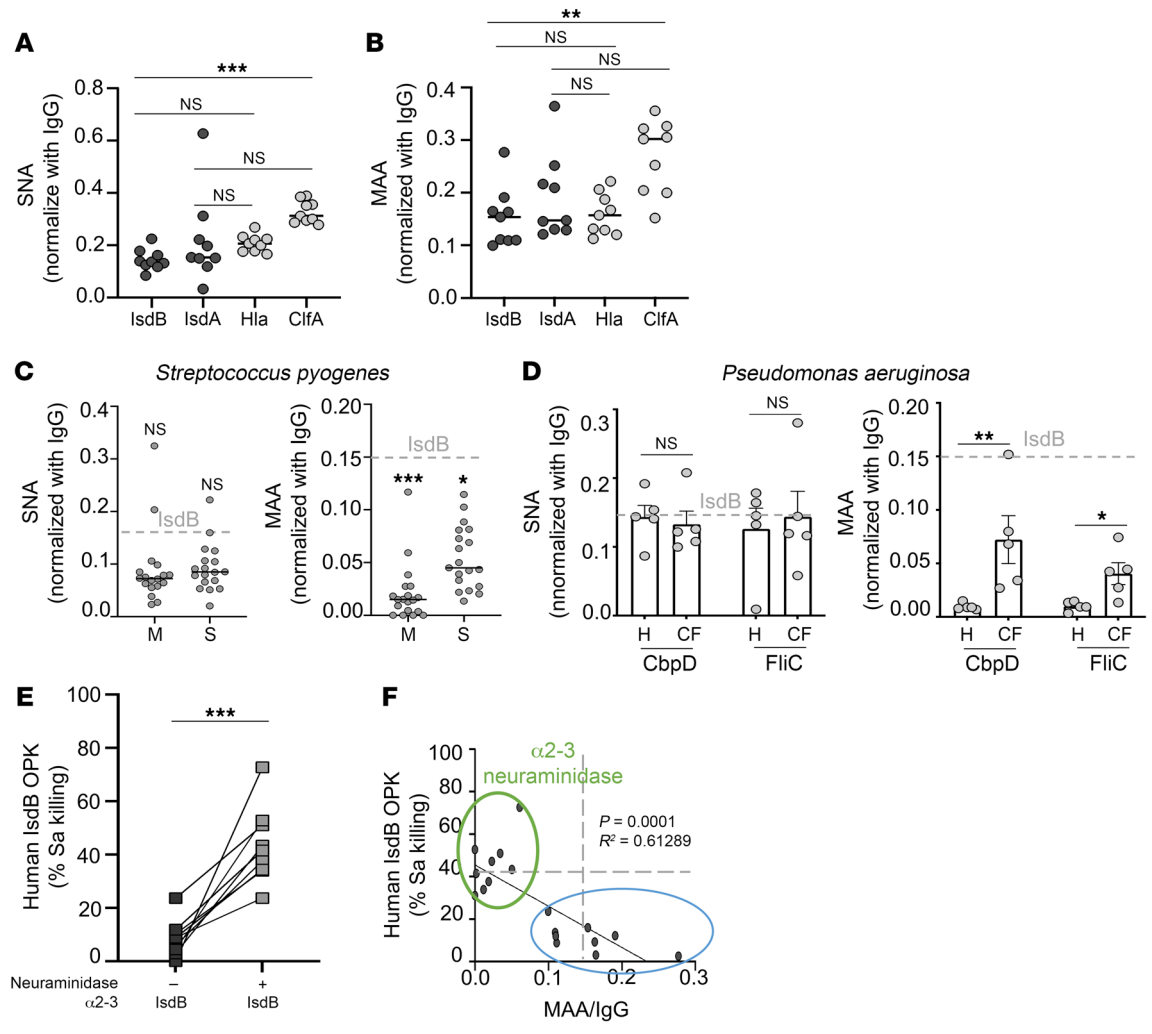
Hyper-α2,3 sialylation of human anti-Sa antibodies. (**A** and **B**) α-2,6 and α-2,3 sialylation of purified human serum Sa antibodies (*n* = 9) as assessed by SNA and MAA lectin binding normalized to IgG titer. (**C**) Sialylation of purified human serum antibodies against M protein (M) or S protein (S) of *Streptococcus pyogenes* (*n* = 18) as assessed by MAA and SNA lectin binding normalized to IgG titer. Gray dashed line indicates median SNA or MAA level of IsdB antibody. (**D**) Sialylation of purified human serum antibodies against *P*. *aeruginosa* CbpD or FliC (*n* = 5) as assessed by MAA and SNA lectin binding normalized to IgG titer. H, healthy normal human; CF, subject with cystic fibrosis. Gray dashed line indicates median SNA or MAA level of IsdB antibody. (**E**) OPK of Sa (LAC) by primary mouse neutrophils in the presence of human IsdB antibodies treated with α2-3 neuraminidase or buffer control from human donors (*n* = 9). (**F**) Correlation between purified human serum IsdB antibody (*n* = 9) binding to MAA lectin and OPK of LAC. Green circle indicates IsdB antibodies treated with α-2,3 neuraminidase. Bars represent group median; each point represents an individual human donor; error bars represent means± SD (**A**–**D**). Each point represents an individual human donor (**E** and **F**). *P < 0.05; **P < 0.01; ***P < 0.001, Student’s *t* test (**C**–**E**), 1-way ANOVA followed by Bonferroni’s multiple-comparison adjustment (**A** and **B**) or linear regression (**F**).

Thus, we purified human antibodies to Sa cell-wall antigens IsdB, IsdA, ClfA, and to Hla (α hemolysin). We measured α2,3 and α2,6 sialylation levels by SNA or MAA binding (Figure 6, A and B). In the absence of a control working human staphylococcal vaccine, we assessed α2,3 and α2,6 sialylation of antibodies against group A *Streptococcus* (GAS) M protein and S protein (Figure 6C) and *Pseudomonas aeruginosa* CbpD or FliC (Figure 6D). GAS infection is not associated with IL-10 release, unlike Sa infection (8). Interestingly, peripheral blood mononuclear cells from cystic fibrosis patients release a higher level of IL-10 compared with healthy subjects, and IL-10 suppressed the subjects’ T cell response to *P*. *aeruginosa* (34). Thus, we included antibodies from both normal and cystic fibrosis subjects in our analysis. Strikingly, the level of α2,3 but not α2,6 sialylation was higher on antibodies against Sa antigens compared with antibodies against *P*. *aeruginosa* and GAS antigens from normal individuals after controlling for IgG concentration (Figure 6, C and D). α2,3 Sialylation was also elevated on antibodies purified from cystic fibrosis subjects compared with antibodies from healthy control subjects (Figure 6D). Treatment of IsdB antibodies with α2,3neuraminidase enhanced OPK of Sa by the human IsdB antibodies (Figure 6, E and F). This proof-of-principle study suggests that findings in mice could be extended to humans but requires further corroboration.

## Discussion

In the current study, we report a mechanism by which the pathobiont Sa induces antiinflammatory cytokine IL-10 to modify sialylation on anti-Sa antibodies, thereby neutralizing their host-protective activity. This nonprotective humoral response is recalled during vaccination, resulting in an ineffective vaccine response (9). Although vaccine generation of de novo anti-IsdB B cells is not quantitatively affected, the B cells acquire a nonprotective phenotype in mice adoptively transferred with Sa-induced B10 cells. This effect is likely due to the direct influence of IL-10 on the surface IL-10R of these B cells. We hypothesize that the induction of IL-10 by Sa is evolutionarily favored, serving as an evasion mechanism to broadly safeguard Sa surface antigens from targeting by the host humoral immunity. Although IL-10 suppression is nonspecific, we also show that it specifically targets anti-Sa vaccines when the vaccine antigens are expressed by Sa during a prior encounter. We propose that antigens expressed on the surface of Sa are likely to induce B or T cell imprints in an IL-10–rich milieu, influencing the development of their protective activities. Although IL-10 does not affect the clonality of the B cell repertoire, a few B cell epitope targets emerge from IL-10 treatment. It remains unclear whether these clones contribute to protective immunity or how they arise as a consequence of IL-10. Irrespective of this uncertainty, α2,3 neuraminidase treatment alone is sufficient to modify efficacy of the vaccine-generated nonprotective antibodies. Although IL-10 induces broad suppression of Sa vaccines — IsdB, IsdA, MntC, and FhuD2 in pathogen-exposed mice — IL-10 has no clear effect on FhuD2 antibody α2,3 sialylation, and it remains unclear whether IL-10’s suppressive effect on FhuD2 vaccines results from the modification of alternative glycans on FhuD2 antibodies or through a direct IL-10 effect on T effector cells.

Relatedly, we have shown that IL-10 modifies the transcriptional activity of additional glycosyl transferases, including terminal galactosyltransferase b4glt1 and several fucosyltransferases. Both fucosylation and galactosylation have been shown to affect antibody binding to FcR (11) and could thereby contribute to IL-10’s control of antibody function. Interestingly, the application of anti–IL-6 antibody at the time of vaccination enhanced sialylation of IsdB antibodies but did not affect ant-Sa vaccine efficacy. Although both IL-6 and IL-10 enhance Fc α2,3 sialylation, we speculate that the 2 cytokines could have different effects on expression of other glycosyl transferases that then leads to different antibody functions. These effects and their impact on FcR and complement binding are likely to be complex and will be addressed in a future study.

IL-10 serves as a potent immunosuppressive cytokine with diverse regulatory functions impacting both innate and adaptive immune responses (35, 36). Its induction is closely linked to Sa virulence factors such as phenol-soluble modulins, SpA, and the staphylococcus toxic shock syndrome toxin (37–39). While our study primarily focuses on the role of IL-10 expressed by B cells, other contributors to IL-10 production include myeloid-derived suppressor cells (MDSCs), T lymphocytes, dendritic cells, M2 macrophages, and neutrophils (22, 40–42). Nonprotective T cell imprints, recalled by Sa vaccines, also emerge as a substantial source of IL-10 (our unpublished observations). Sa induces IL-10 in conjunction with colonization, biofilm formation, and infections (8, 21, 22). During colonization, Sa triggers the production of IL-27 and IL-10 through TLR2 signaling, inhibiting protective T cell responses and creating an immunosuppressive milieu (21). IL-10 induction during Sa infection is associated with the inhibition of protective CD4^+^ T cell development, allowing for Sa reinfections (8). IL-10–suppressive effects on innate immunity further contribute to repressed anti-Sa immunity.

Notably, IL-10 is strongly associated with mortality in Sa bacteremic patients (43). The human IsdB vaccine trial was terminated early because of a 5-fold increase in mortality in vaccinated subjects (20), although IL-10 levels were not measured in vaccine recipients. Our current study provides evidence that IsdB vaccination in pathogen-exposed hosts is associated with higher IL-10 levels and reduced IL-1α, INF-γ, and IL-6, independently of CFU burden. Therefore, the abundance of IL-10 and reduced protective inflammatory cytokines released to engage the pathogen, in association with vaccine-recalled T or B cell imprints, could potentially explain the high mortality associated with the IsdB vaccine trial. Although we did not observe mortality differences in association with IsdB vaccination in Sa-exposed mice under our experimental conditions, more rigorous studies are warranted to address this human-relevant finding.

Antibody glycosylation influences pathology or protection against infectious diseases (17). In tuberculosis, individuals with active tuberculosis have increased afucosylated antibodies, leading to increased FcγRIIIa binding and enhanced capacity to kill *Mycobacterium tuberculosis* intracellularly (44). In a model of pregnancy-induced antibody protection against *Listeria*, deacetylation of terminal sialic acid residues on antibody N-linked glycans protected neonates by engaging CD22, inhibiting IL-10 production by B cells (45). We identified increased α2,3 sialylation in mouse and potentially human antibodies as evidence of nonprotective antibodies. This finding could serve as a potential marker for evaluating the efficacy of Sa vaccines, a crucial aspect that is currently lacking. Despite abundant evidence indicating the clear role of antibody glycan in coordinating innate and adaptive immune responses, the upstream immune control of antibody glycosylation is generally not well studied, especially in infectious diseases. In autoantibody-driven rheumatoid arthritis, T_H_17 cells regulated the expression of β-galactoside α2,6-sialyltransferase 1 through IL-22 and IL-21, which induced glycan changes in autoantibodies coinciding with the inflammatory phase of arthritis (18). Another study interrogated the influence of selective cytokines on human CD19^+^ B cell–derived antibody-secreting cells, demonstrating a positive influence of IFN-γ on galactose level on IgG and augmentation of IgG sialylation by IL-21 and IL-17A (46).

Conversely, in a model of chronic viral infection, IL-10 induction of glycosyltransferase *Mgat5* enhanced N-glycan branching on the surface of CD8^+^ T cells, reducing the sensitivity of the immune cell to antigens (19). Here, we demonstrate a direct influence of IL-10 in antibody a2,3 sialylation, reducing the protective efficacy of antibodies. Unlike the host’s protective response against *Listeria*, we suggest that IL-10 is elicited by Sa to maintain its pathobiont lifestyle. Conversely, the profound reversal of antibody function with IL-10–neutralizing interventions points to an opportunity to modify antibody biologic function through cytokine neutralization or selective adjuvancy. For example, low IgG Fc α2,6sialylation can be induced by adjuvants that promote follicular helper T (T_FH_) cell-inducing cytokine IL-6, IL-27 receptor–dependent IFN-γ^+^ T_FH1_ cells, or IL-6/IL-23–dependent IL-17A^+^ T_FH17_ cells (47). Modulating antibody glycoforms represents an area of research that could improve on the currently suboptimal vaccination strategies (48).

In summary, we describe a pathobiont-mediated immune-suppressive mechanism that broadly diminishes the efficacy of Sa vaccines by modulating antibody sialylation in mice. Our preliminary human data suggest a similar mechanism of antibody nonresponsiveness. Particularly intriguing are the data on anti-pseudomonas antibodies, revealing high α2,3 sialylation associated with cystic fibrosis compared with healthy individuals. Antibodies from cystic fibrosis subjects are also nonfunctional (49), and there is a concurrent induction of high IL-10 levels from cystic fibrosis peripheral blood mononuclear cells compared with cells from normal subjects (34). These observations reinforce the link between sialylation and IL-10 in pathobionts other than Sa. These discoveries should prompt a more comprehensive assessment of human anti-Sa antibodies, extending the investigations to include antibodies against other pathobionts and antibodies from unsuccessful human vaccine trials. Notably, IL-10 is commonly induced by commensals and pathobionts to facilitate coexistence with the mammalian host (50). Consequently, the suppressive mechanism observed in Sa vaccines may have relevance to failed vaccines targeting other pathobionts, including ESKAPE organisms. Conversely, exploring solutions based on cytokines or adjuvants to remodel antibody glycan structures could offer potential avenues for restoring Sa vaccine protection.

### Limitations of the study.

Our study primarily focused on a mouse model, and IsdB antibodies before and after human vaccination were not available for analysis (V710; Merck Sharp & Dohme Corp.) (20). As such, our findings provide a conceptual framework for understanding staphylococcal vaccine interference. Caution should be exercised in extrapolating the data to human trials.

## Methods

### Sex as a biological variable.

Our prior work has established the negative impact of immune imprinting on IsdB vaccination in both male and female mice (9). The current study expands on the role of IL-10 on antibody sialylation, which is only studied in female mice. This is an acknowledged flaw of the study. For the analyses of sialylation of human antibodies, sera from both male and female subjects were used.

### Murine models of Sa infection.

C57BL/6 and CD45.1 (Ly5.1) mice were purchased from Charles River Laboratories. CD19cre [B6.129P2(C)-Cd19^tm1(cre)Cgn^/J] and IL-10Rαflox mice [B6(SJL)-​IL-10ra^tm1.1Tlg^/J] were purchased from The Jackson Laboratory. CD19cre mice were crossed with IL-10Rαflox mice to generate *CD19cre-IL-10Rα^fl/fl^* mice. *Sa* Becker and isogenic IsdB/HarA deletion mutant were gifts from S. Secore (Merck). Overnight Sa cultures were diluted 1:200 in Todd Hewitt broth (THB) and grown to an optical density of 0.8. Unless otherwise stated, 6- to 8-week-old female mice were administered 1–2 × 10^7^ CFU of LAC (USA300) i.p. for each Sa challenge. Spleen and kidneys were harvested 24 hours after the last infection, homogenized in PBS, and plated on THB agar plates for CFU enumeration. Another Sa inoculum used was 4 × 10^7^ CFU of Becker (WT or IsdB/HarA mutant) i.p.

### Cloning and protein expression.

The *IsdB* gene was amplified from LAC using primers: 5′IsdB (5′-GGTCGCGGATCCAACAAACAGCAAAAAGAATTT-3′) and 3′IsdB (5′-GGTGGTGCTCGAGTTTAGTTTTTACGTTTTCTAGGTAATAC-3′). The PCR product was cloned into pET28 expression vector (Novagen) and expressed as described previously with some modifications. Briefly, IsdB-expressing plasmids were used to transform *E*. *coli* BL21 (DE3) cells (NEB) to produce a His-tagged protein with 1 mM of isopropyl-β-d-thiogalactoside (IPTG) for 2 hours. Recombinant *E*. *coli* was centrifuged and suspended in lysis buffer (50 mM Tris-HCl [pH 8.0], 0.1 M NaCl, 2 mM MgCl_2_, 10 mM imidazole, 0.1% Tween 80, 1% Triton X-100, PMSF, lysozyme [2 mg/mL]). His-tagged IsdB was purified from the clarified lysate by His60 Ni Superflow Resin (Takara) chromatography. The column was washed with 20 mM Tris-HCl (pH 8.0), 150 mM NaCl, and 0.1% Tween 80, and His-tagged IsdB was eluted with 300 mM imidazole, 20 mM Tris-HCl (pH 7.5), 150 mM NaCl, and 0.1% Tween 80 (26). The fliC gene was amplified from *P*. *aeruginosa* PAO1 strain using primers 5′ fliC (5′-CTCGGATCCCACTCAGCGCAACC-3′) and 3′fliC (5′-ACGAAGCTTGCAGCAGGCTCAG-3′) (51). The PCR product was cloned into the pET28 expression vector and expressed as described previously (1). The recombinant S protein, M protein, and CbpD were cloned and purified as described (52–54).

### Immunization with IsdB vaccine and antibody purification.

Mice were immunized i.p. or i.m. 3 times with IsdB (75 μg, 50 μg, and 50 μg) plus aluminum hydroxide (alum, InvivoGen) (450 μg per dose) or with aluminum hydroxide alone at 7-day intervals. Mouse sera were screened for reactivity to IsdB by ELISA. IsdB-specific antibodies were purified from mouse sera using immobilized IsdB agarose columns (NHS-activated agarose, Thermo Fisher Scientific).

### Opsonophagocytosis assay.

Opsonophagocytosis assay was performed as described. Mouse neutrophils were isolated from bone marrow by MojoSort Mouse Neutrophil Isolation Kit (BioLegend). Overnight culture of *Sa* LAC was subcultured 1:200 in THB and grown to an optical density of 0.6. Sa was washed, resuspended in PBS, and incubated with mouse sera at 37°C for 20 minutes, then added to 10^5^ mouse neutrophils at a multiplicity of infection (MOI) of 1:0.5 in the presence of 2% normal mouse serum. Following incubation at 37°C for 1 hour with agitation at 200 rpm, samples were plated on THB agar plates for CFU enumeration (55).

### ELISA and multiplex bead assays.

Serum IL-10 was measured using the ELISA MAX Deluxe Set Mouse IL-10 Kit (BioLegend, 431426). Serum inflammatory cytokines were measured with the LEGENDplex Mouse Inflammation Panel (BioLegend, 740446). IsdB-specific antibody levels in human and mouse sera were measured by ELISA as described. Briefly, sera were serially diluted in PBS containing 1% BSA and added to 96-well plates coated with recombinant IsdB (1 μg/mL). Bound antibodies were detected by horseradish peroxidase–conjugated (HRP-conjugated) goat anti-mouse IgG or anti-mouse IgM (BioLegend).

IsdB-, M protein–, S protein–, CbpD-, and FliC-specific antibody levels in human and mouse sera were measured by ELISA as described. Briefly, high binding microtiter plates (Corning) were coated with 1 μg/well recombinant His-tagged IsdB, M protein, S protein, CbpD, and FliC in PBS and incubated overnight at 4°C. Sera were serially diluted in PBS containing 1% BSA and added to the 96-well plates coated with recombinant IsdB, CbpD, and Flic. Bound antibodies were detected by HRP-conjugated goat anti-mouse IgG, anti-mouse IgM (BioLegend), and HRP-conjugated donkey anti-human IgG (clone poly24109, BioLegend 410902, dilution 1:5,000) using the 3,3ʹ,5,5ʹ-Tetramethylbenzidine (TMB) substrate set (OptEIA).

### Lectin-ELISA.

Lectin-ELISA was described previously (56). Briefly, high-binding microtiter plates were coated with 10 μg/mL protein G (Sigma-Aldrich, 19459-5MG-F) in TBS at 4°C overnight and blocked with TBS containing 1% BSA for 1 hour; 1 μg of purified antibody was diluted in 100 μL of TBS-BSA and incubated for 3 hours at 37°C. The plates were then incubated with 100 μL of biotinylated SNA (0.25 μg/mL dilution, Vector Laboratories, B-1305-2), MAA (4 μg/mL dilution, Vector Laboratories, B-1265-1), ECA (1 μg/mL dilution, Vector Laboratories, B-1145-5), or PHA-L (1 μg/mL dilution, Vector Laboratories, B-1115-2) in TBS-T (Tris-buffered saline with 0.05% Tween-20) for 60 minutes at 37°C. Bound lectins were detected by HRP-conjugated Avidin (BioLegend, 405103) using the TMB substrate set.

Sialylation of purified human serum antibodies against recombinant bacterial proteins was assessed by MAA and SNA lectin-binding antibodies. Briefly, high-binding microtiter plates were coated with 1 μg/well recombinant protein in PBS and incubated overnight at 4°C. The plates were blocked with PBS containing 1% BSA for 1 hour at room temperature. Next, sera were serially diluted (1:100) in PBS and added to the coated wells. The plates were washed with TBS supplemented with 0.05% (v/v) Tween 20 (MilliporeSigma) (TBST). The level of sialylation was identified with diluted biotinylated SNA and MMA antibodies (100 μL/well) in TBS-BSA and incubated for 1 hour at 37°C. The bound lectins were detected by incubation of the plate for 30 minutes at room temperature with diluted HRP-conjugated avidin (1:1000) in TBS-BSA. To measure sialylation of IgG subclasses, 96-well plates were coated with capture antibody toward IgG1 (BioLegend, 406601), IgG2b (BioLegend, 406701), IgG2c (Bio-Rad, STAR135A) or IgG3 (BioLegend, 406802) in carbonate coating buffer at 4°C overnight and blocked with TBS containing 1% BSA for 1 hour; 1 μg of purified antibody was diluted in 100 μL of TBS-BSA and incubated for 3 hours at 37°C. The bound antibodies were incubated with lectin to measure sialylation.

### Flow cytometric analysis.

Splenocytes were harvested and suspended at a density of 5 × 10^6^ cells/mL in PBS with 2% FBS; 100 μL of cell suspension was used for each staining experiment. All fluorophore-conjugated antibodies were purchased from BioLegend. The following antibodies were used at 1:200 dilution: anti-B220-allophycocyanin (APC) (RA3-6B2, 103212), anti-CD21-fluorescein (FITC) (7E9, 123408), anti-CD23–phycoerythrin (PE) (B3B4, 101608), anti-CD1d–PerCP/Cy5.5 (1B1, 123513), anti-CD5-PE (53-7.3, 100607), anti–GL-7–PerCP/Cy5.5 (GL7, 144609), anti-CD95–PE (SA367H8, 152607), anti-CD45.1–FITC (A20, 110706), and anti-CD45.2–PerCP/Cy5.5 (104, 109828). Data were acquired on the BD FACS Canto system using BD FACSDiva software, version 7.0, and analyzed using FlowJo (NIH).

### Adoptive transfer of B lymphocytes, sera, or purified antibodies.

Splenic CD19^+^ B cells (MojoSort Mouse Pan B Cell Isolation II Kit, 480088, BioLegend) or CD45.1^+^ cells (MojoSort Mouse CD45.1 Selection Kit, BioLegend, 480118) were isolated using kits following instructions provided by the manufacturer; 2 × 10^7^ B cells were injected i.v. into recipient mice. IsdB immune sera were generated by Sa infection or immunization as described above. Human sera were obtained from anonymized adult human volunteers. IsdB-specific antibodies were purified from mouse sera using immobilized IsdB agarose columns (NHS-activated agarose, Thermo Fisher Scientific).

For neuraminidase treatment, purified antibodies were treated with *Streptococcus pneumoniae* α2-3 neuraminidase S (8,000 units/mL; New England Biolabs, P0743S) or buffer alone at 4°C for 16 hours, followed by PBS washes.

### In vivo neutralization of cytokines, B10 cell depletion, and administration of recombinant IL-10.

All antibodies for in vivo experiments were purchased from Bio X Cell. For neutralization of cytokines, 100 μg of IL-6– (BE0046), IL-10– (BE0049), IL-12– (BE0052), or IL-23–neutralizing (BE0313) antibodies were administered i.p. 3 times at 24 hours before, 4 hours before, and 16 hours after each vaccination. For depletion of B10 cells, 300 μg of anti-mouse CD22 antibody (BE0011) was administrated i.p. 16 hours before each vaccination.

Recombinant IL-10 treatment consisted of administration of 100 ng of recombinant IL-10 (Biolegend, 575806) i.p. 3 times at 16 hours before, 8 hours after, and 16 hours after each vaccination.

### RT-PCR and primers.

RNA from cells was isolated using the RNeasy Mini Kit (QIAGEN, 74104) following instructions provided by the manufacturer. cDNA was prepared using the Verso cDNA Synthesis Kit (Thermo Fisher Scientific, AB1453A). Real-time PCR was performed using SYBR Select Master Mix (Thermo Fisher Scientific, 4472942) and run on the CFX96 Real-Time System (Bio-Rad). cDNA expression was analyzed by the ΔCt method normalized to values of mGAPDH. The following primers were used: mST3GAL1: forward 5′-ATCTACCACCCAGCCTTCA-3′and reverse 5′-TGTTCTCCCAGTAATGGTGC-3′; mST3GAL2: forward 5′-GAGGGGCTTTTGGGGAGAAA-3′ and reverse 5′-TGTAGCATCATCCACCACCG-3′; mST3GAL3: forward 5′- AACTTTTCCGAGGGAGCTTG-3′ and reverse 5′-TAGCCCACTTGCGAAAGGAG-3′; mST3GAL4: forward 5′-TGGGTAAAGACGCCATCCAC-3′ and reverse 5′-TCGAGGCTCTTTATGCTCTCAG-3′; mST6GAL1: forward 5′-CCTTCAACACCACTGAATGG-3′ and reverse 5′-TAAACCTCAGGACCGCATC-3′; mST6GAL2: forward 5′-CTGCGCAGTTGTCATGTCTG-3′ and reverse 5′-TTTCTCATAGCCACGTGTAGGG-3′; mMgat5: forward 5′-GTCTCTGGCGGAGAAACAAA-3′ and reverse 5′-TGCTGTCTCCGCAATCTTG-3′; mFut4: forward 5′-CAGCCTGCGCTTCAACATC-3′ and reverse 5′-CGCCTTATCCGTGCGTTCT-3′; mFut8: forward 5′-AGACCAGAAATGGTCTGGGGAA-3′ and reverse 5′-CCAATCACCTGCTCCATCTGTC-3′; mFut11: forward 5′-TAACTTGGAAGACTGCGTTACTG-3′ and reverse 5′-GGCTGAGATACTAGCTCCATACC-3′; mB4GalT1: forward 5′-CGGCAGGAGCATCTCAAATA-3′ and reverse 5′-CAGCCTGATTGATGACGTAGAT-3′; and mGAPDH: forward 5′-TTAGCACCCCTGGCCAAGG-3′ and reverse 5′-CTTACTCCTTGGAGGCCAT-3′.

### ChIP.

Splenic B cells were cultured with 10 ng/mL of recombinant IL-10 or PBS buffer control for 16 hours. ChIP was performed according to the manufacturer’s protocol (SimpleChIP, Cell Signaling Technology, 4472942). Briefly, cells were treated with formaldehyde to crosslink proteins to DNA and then lysis, and subjected to sonication using a Sonic Dismembrator (Fisher Scientific) to obtain chromatin fragments; 200 μg of fragmented chromatin was incubated with 10 μg of STAT3 antibody (Cell Signaling Technology, 9132L) and incubated with the protein G magnetic beads. Eluted chromatin from the beads and crosslinks was reversed by 16 hours at 65°C. DNA was purified by spin columns. Real-time PCR was performed using SYBR Select Master Mix (Thermo Fisher Scientific, 4472942) and run on the CFX96 Real-Time System (Bio-Rad). The result was analyzed by the ΔCt method normalized to values of buffer control group. The following primers were used: mMgat5_1-1: forward 5′-CCCTGACTTCCCTCTGTAATG-3′and reverse 5′-GGGCAGAATTCT AGTTTCCTCT-3′; mMgat5_1-2: forward 5′-GAACTAGCCACCACTAGTTCATT-3′and reverse 5′-CTCCACAGGAACATGTACATACC-3′; mMgat5_2-1: forward 5′-ACACCACAGGTGTGTTCTATG-3′and reverse 5′-CAATTTAGTTCCTCTTCTGCGTTT-3′; mMgat5_2-2: forward 5′-TTTGGCTTGCAAGGAAGAATG-3′and reverse 5′-GTGTCAGAGACAGTCCAAGTG-3′; mSt3gal2_1-1: forward 5′-GTGGCACATGCCTTTAATTCC-3′and reverse 5′-CTGGCTGTCCTAGAACTCATTC-3′; mSt3gal2_1-2: forward 5′-GGCAGGTGGATTTCTGACTT-3′and reverse 5′-GACAGGGTTTCTCTGTGTAGC-3′; mSt3gal3_1-1: forward 5′-CGTTCGTGGAAGAGAGGAAA-3′and reverse 5′-CACGAGATTATCCGGTCCTTTAG-3′; mSt3gal3_1-2: forward 5′-CCTTGCTAAAGGACCGGATAAT-3′and reverse 5′-TAGCTTGGCAGTAGTACGTTTG-3′; mSt3gal4_1-1: forward 5′-GCTACAGACTTCAGGGTATCAAG-3′and reverse 5′-GAGTGATCTCATGGTGCTACG-3′; mSt3gal4_1-2: forward 5′-GGCCTTGGGTTTGCTATCTA-3′and reverse 5′-TCTGTGAAGTGAAAGGCTGAT-3′; mSt3gal4_2-1: forward 5′-CTGGGTCTTACTTGCTCCTTT-3′and reverse 5′-GCACCTCAGCAGTGTTATCT-3′; mSt3gal4_2-2: forward 5′-ACTGTGGGTGGGAGATAACA-3′and reverse 5′-GTGTGCAAGCCTGAACTCATA-3′; mSt6gal2_1-1: forward 5′-CCTCAACTGCTGGTTCTACTC-3′and reverse 5′-TGCCATAACCCATAGCCATAC-3′; mSt6gal2_2-1: forward 5′-GAGTCCATCGTGGTGAATTGT-3′and reverse 5′-CTAAGAACTGCCTGAAGGGATG-3′; mSt6gal2_2-2: forward 5′-CATCCCTTCAGGCAGTTCTT-3′and reverse 5′-GGGAAACTAAACCCACAGAGT-3′; mSt6gal2_3-1: forward 5′-GGTTTAGTTTCCCGGTTGTTAAAT-3′and reverse 5′-TGGAGTGCCTACTATGTGTTTG-3′; mSt6gal2_3-2: forward 5′-GTAGGTAACACTCACATCCAAGG-3′and reverse 5′-GTCACTGCAACCCTCCTATTT-3′; mSt6gal2_3-3: forward 5′-GGCATTAGCACTTGAACACAC-3′and reverse 5′-CAACTCCATCCATCCATCCAA-3′; mFut4_1-1: forward 5′-GGCCTCTAGGATAAGCACATAAC-3′and reverse 5′-GCTGAATGCACACCTCTCA-3′; mFut4_1-2: forward 5′-ATTCAGCTCCAGAAACCTTACA-3′and reverse 5′-GAGAGAGCTGGTGTTTCCTAAG-3′; mFut4_2-1: forward 5′-TGAGATTTGACGCCCTCTTC-3′and reverse 5′-TGCAAGCCTACCATCAGTATC-3′; mFut4 _2-2: forward 5′-GGAAAGGCCACTGACACATAA-3′and reverse 5′-GCTGGAATGTAGTCAGGACAA-A-3′; mFut8_1-1: forward 5′-TGGCTATCCCTGTCTCATAGT-3′and reverse 5′-TTCCCAGAACAGGAGAGAATTG-3′; and mFut8_1-2: forward 5′-GGCTAAGTGATAGTAACTACTGTAAGG-3′and reverse 5′-GCCAAACTATGAGACAGGGATAG-3′.

### Preparation of single-cell sequencing.

Splenocytes from IsdB-immunized or LAC-infected mice were incubated with PE–labeled IsdB and APC-conjugated anti-mouse CD45R/B220. IsdB^+^B220^+^ cells were sorted by FACSAria II (BD) and subjected to single-cell preparation by using a Single Cell 5′ Library and Gel Bead Kit (10X Genomics) and Chromium Single Cell A Chip Kit (10X Genomics). The cell suspension was loaded onto a chromium single-cell controller to generate single-cell gel beads in the emulsion (GEMs) according to the manufacturer’s protocol (10X Genomics). scRNA-Seq libraries were constructed using a Chromium Single Cell V(D)J Enrichment Kit, Mouse B Cell following instructions provided by the manufacturer (10X Genomics). The libraries were sequenced using an Illumina NovaSeq 6000 sequencer with a paired-end 150-bp (PE150) reading strategy (performed by Institute for Genomic Medicine, UCSD).

### Identification of clones and comparisons of clonotypes.

The clonal groups were identified by the *R* package – *scRepertoire* (57) based on paired heavy and light chains. To determine clonal groups, we first used the filtered contig annotation obtained from the results performed using the Cell Ranger Single-Cell Software Suites (http://software.10xgenomics.com/single-cell/overview/welcome). Then for the cells with high quality paired heavy and light chains that were sequenced, clones were assigned based on strict definition of clonotype using the *CTstrict()* function that considers clonally related 2 sequences with identical V gene usage and greater than 85% normalized Levenshtein distance of the nucleotide sequence. The clonotype changes between samples were visualized by the *compareClonotypes()* function with the clones called by amino acid sequence of the CDR3 region (57–63).

### N-glycan analysis by ultra pressure liquid chromatography with fluorescence detector.

Purified N-glycans were dried and tagged with procainamide fluorophore and analyzed using UPLC-FL. Briefly, tagging reagent was prepared by dissolving 14.0 mg of procainamide in 100 μL of DMSO: acetic acid (HOAc) mixture (65:35 v/v), followed by transferring the procainamide solution to 12 mg of 2-picoline borane taken in a separate tube; 10 μL of tagging reagent was added to the known amount of N-glycan, sonicated for 30 seconds, and incubated at 65°C for 2.5 hours on a heating block covered with aluminum foil to protect from light. A known amount of N-glycans was mixed with 1 μL of 250 mM ammonium formate and 15 μL of acetonitrile using micropipette and taken in autosampler vials for N-glycan analysis using the UPLC-FL system. A gradient mixture of eluent-A (100 mM ammonium formate, pH 4.5) and eluent-B (acetonitrile) was used as a running buffer for UPLC. For profiling of N-glycans acquity UPLC BEH Glycan Analysis Column (Waters, 2.1 mm × 150 mm, 1.7 μm) with Acquity UPLC Glycan BEH Amide VanGuard Pre-Column (Waters, 2.1 mm × 5 mm, 1.7 μm) was used at variable flow rate. The column and autosampler temperature were set at 60°C and 10°C, respectively. The injection volume was kept at 10 μL. The fluorescence detector was set with excitation wavelength of 310 nm and emission wavelength at 370 nm with photomultiplier tube (PMT) gain of 5 (64–67).

### MALDI–mass spectrometry analysis of N-glycans.

Purified N-glycans were permethylated and analyzed by MALDI-Tof/Tof mass spectrometry (Bruker, AutoFlex) in positive reflectron mode (64, 68). Briefly, N-glycans were dried completely and redissolved in anhydrous DMSO, followed by permethylation using NaOH slurry in anhydrous DMSO and CH3I. Permethylated glycans were extracted with chloroform and dried completely using dry nitrogen flush. The permethylated N-glycans were dissolved in mass spectrometry grade MeOH and then mixed with Super-DHB (MALDI matrix) in 1:1 (v/v) ratio before spotting on MALDI plate, and 1 μL of sample was spotted and allowed it to crystallize at room temperature prior to acquiring mass spectra. All MALDI mass spectral data on permethylated N-glycans were acquired in positive, reflectron mode. Finally, the mass spectral data were analyzed and plausible N-glycan structures were annotated using the GlycoWork Bench software selecting CFG database, version 2.1 (64, 68).

### Statistics.

All statistical details of experiments, including the statistical tests used, exact value of *n*, what *n* represents, definition of center, and dispersion and precision measures can be found in the figure legends. Two-group analysis used unpaired Student’s *t* test (2-tailed tests). In vivo experiments were analyzed using nonparametric Mann-Whitney *U* test. Comparisons of multiple groups were performed using 1-way ANOVA, with Kruskal-Wallis test in the case of missing normality. Data were presented as mean ± SD, unless otherwise indicated. Statistical significance was assigned as *P* ≤ 0.001; *P* ≤ 0.01; *P* ≤ 0.05; *P* > 0.05; and NS (not significant). Analyses were performed using GraphPad Prism 10.

### Study approval.

Mouse studies were reviewed and approved by the Institutional Animal Care and Use Committee. Mouse experiments were conducted in accordance with recommendations listed in the Animal Care Program at UCSD and Cedars-Sinai Medical Center’s regulations and recommendations on animal experiments. Blood was collected from several healthy volunteers and cystic fibrosis patients in accordance with the Helsinki Declaration’s ethical principles. All blood donors signed a written informed consent form. The collections of blood samples were approved by the UC San Diego Human Research Protection Program under IRB protocol numbers 131002 and 160078, respectively.

### Data availability.

Any data generated or analyzed during this study, associated protocols, materials within the manuscript, and public databases (NCBI’s Gene Expression Omnibus [GEO] GSE193543) are included in the article and related supplementary information or are available from the corresponding author. Source data are provided with this paper. The codes used for the analysis were not designed to be made public but can be requested from the corresponding author. Values for all data points in graphs are reported in the Supporting Data Values file.

## Author contributions

CMT and GYL designed all the experiments and interpreted results. CMT carried out the in vitro and in vivo experiments from Figure 1, Supplemental Figure 1, Figure 2, Supplemental Figure 2, Supplemental Figure 3, Supplemental Figure 4, Figure 3, A–C, Supplemental Figure 5, B–D, Supplemental Figure 6, Figure 4, A and B and E–G, Supplemental Figure 7, A and E–I, Figure 5, Supplemental Figure 8, Supplemental Figure 9, and Figure 6, C, E, and F with support from CG. JRC performed experiments from Figure 6, A, B, and F. BC performed glycan analysis of experiments associated with Figure 3D and Supplemental Figure 5F. ES and XD performed RT-PCR from Figure 4, C and D, and Supplemental Figure 5, B–D. AWTC, HL, and NEL performed single-cell analysis for Supplemental Figure 5A. VN and FA performed and analyzed the experiment in Figure 6D. BL performed recombinant protein purification. IAH contributed to Figure 3, D and E, and Supplemental Figure 5, E and F, and provided the FliC expressing plasmid. TCC and AMR provided recombinant M protein. CJD provided serum samples from cystic fibrosis subjects. IHW and DJG provided recombinant S protein. CMT and GYL wrote the manuscript with all other authors providing significant input.

## Supplementary Material

Supplemental data

Supporting data values

## Version 1 12/16/2024

Electronic publication
