# Supplementary material for: Pathobiont-driven antibody sialylation through IL-10 undermines vaccination
Source: J Clin Invest. 2024 Dec 16;134(24):e179563. doi: 10.1172/JCI179563 (PMC11645145; doi:10.1172/JCI179563)
Supplement: Supplemental data [file jci-134-179563-s192.pdf]

Pathobiont-driven antibody sialylation through IL-10 undermines vaccination

Chih-Ming Tsai[1,*], Irshad A. Hajam[1], J.R. Caldera[1], Austin W.T. Chiang[2], Cesia Gonzalez[1], Xin Du[1], Biswa Choudhruy[3], Haining Li[4], Emi Suzuki[10], Fatemeh Askarian[5], Ty'Tianna Clark[6], Brian Lin[1], Igor Wierzbicki[7], Angelica M. Riestra[6], Conrad J. Douglas[8], David J. Gonzalez[7], Victor Nizet[5,7], Nathan E. Lewis[4,5], George Y. Liu[1,9,*]

[1]Division of Infectious Diseases, Department of Pediatrics, University of California, La Jolla, CA 92093, USA

[2]Immunology Center of Georgia, Augusta University, Augusta, GA 30912, USA; Department of Medicine, Augusta University, Augusta, GA 30912, USA

[3]Glycobiology Research and Training Center, University of California San Diego, La Jolla, CA 92093, USA

[4]Department of Bioengineering, University of California, La Jolla, CA 92093, USA.

[5]Division of gastroenterology, Department of Pediatrics, University of California San Diego, La Jolla, CA 92037, USA

[6]Division of gastroenterology, Rady Children's Hospital, San Diego, CA 92123, USA

[7]Division of Host-Microbe Systems & Therapeutics, Department of Pediatrics, UC San Diego School of Medicine, La Jolla, CA 92093, USA.

[8]Department of Biology, San Diego State University, San Diego, CA 92182, USA

[9]Skaggs School of Pharmacy and Pharmaceutical Sciences, University of California, La Jolla, CA 92093, USA

[10]Division of Pulmonary, Critical Care and Sleep Medicine, University of California San Diego, La Jolla, CA 92037, USA

[11]Division of Infectious Diseases, Rady Children's Hospital, San Diego, CA 92123, USA

*Address correspondence to:

George Y. Liu, Division of Infectious Diseases, Department of Pediatrics, UCSD, BRF2 Room 4119, Gilman Drive, Mail Code 0760, La Jolla, CA 92093, USA. Phone: 858.2465830; gyliu@health.ucsd.edu.

or

Chih-Ming Tsai, Division of Infectious Diseases, Department of Pediatrics, UCSD, BRF2 Room 4220 Bay C, Gilman Drive, Mail Code 0760, La Jolla, CA 92093, USA. Phone: 858.8225993; c8tsai@health.ucsd.edu.

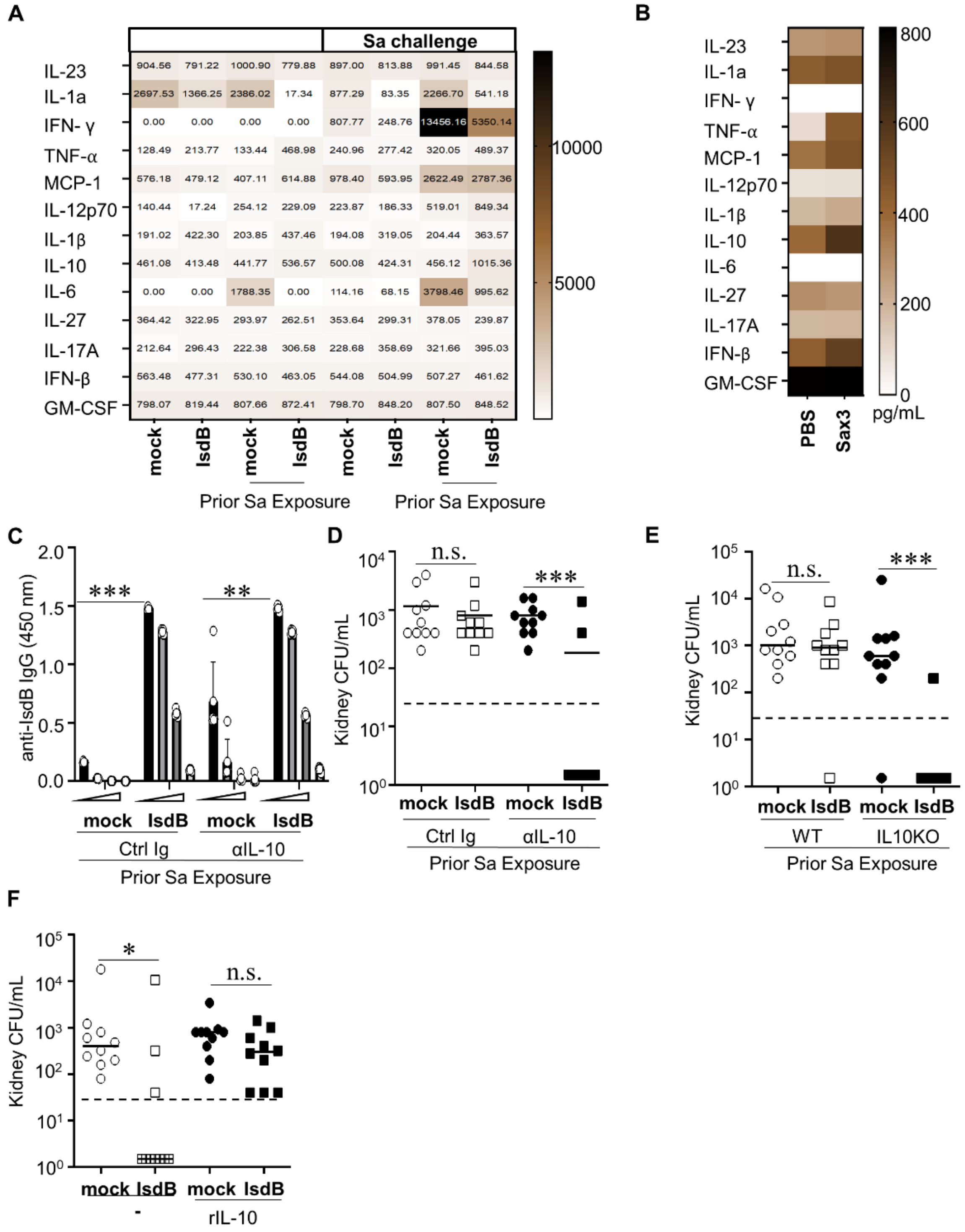

Figure S1 Sa-induced IL-10 abrogates staph vaccine efficacy

(A) Effect of IsdB vaccination on serum cytokines, measured by multiplex cytokine assay. Vaccination was performed on naïve or Sa-infected mice as in Figure 1A. Sera were analyzed 7 d after final vaccination or 1 d after a final Sa challenge.

(B) Effect of Sa infection (x3) on murine serum cytokines, measured by multiplex cytokine assay 7 d after last infection.

(C) Serum IsdB antibody titer in Sa-infected mice treated with IL-10-neutralizing antibody (αIL-10) or control IgG (Ctrl Ig) during immunization as in Figure 1D.

(D) IL-10-neutralizing antibody restores IsdB antibody protection. Mice were infected as in Figure 1D (in the presence of αIL-10 or Ctrl Ig at the time of IsdB vaccination). Serum was adoptively transferred 7 days after vaccination to assess for anti-Sa immunity by LAC challenge. Kidney CFU were measured (n=10 per mouse group).

(E) IsdB vaccination induced antibodies are protective in IL-10-KO mice. C57BL/6 mice (WT) or congenic IL-10 knock out mice (IL-$10^{-/-}$) mice were infected as in Figure 1A. Serum was adoptively transferred 7 d after vaccination to assess for anti-Sa immunity by LAC challenge. Kidney CFU were measured (n=10 per mouse group).

(F) Recombinant IL-10 limits IsdB vaccine efficacy. Naïve mice were administered 100 ng of recombinant IL-10 or control buffer with IsdB vaccination as in Figure 1A. Serum from immunized mice were assessed for anti-Sa immunity after adoptive transfer into naïve mice. Kidney CFU were measured after final Sa challenge (n=10 per mouse group).

Bars represent group mean; error bars represent means ± SD (C). Bar represents group median; each point represents an individual mouse; dashed lines indicate the limit of detection (D to F). n.s., not significant, $*p < 0.05$, $**p < 0.01$, and $***p < 0.001$; one-way ANOVA followed by Bonferroni multiple comparison adjustment (A to F).

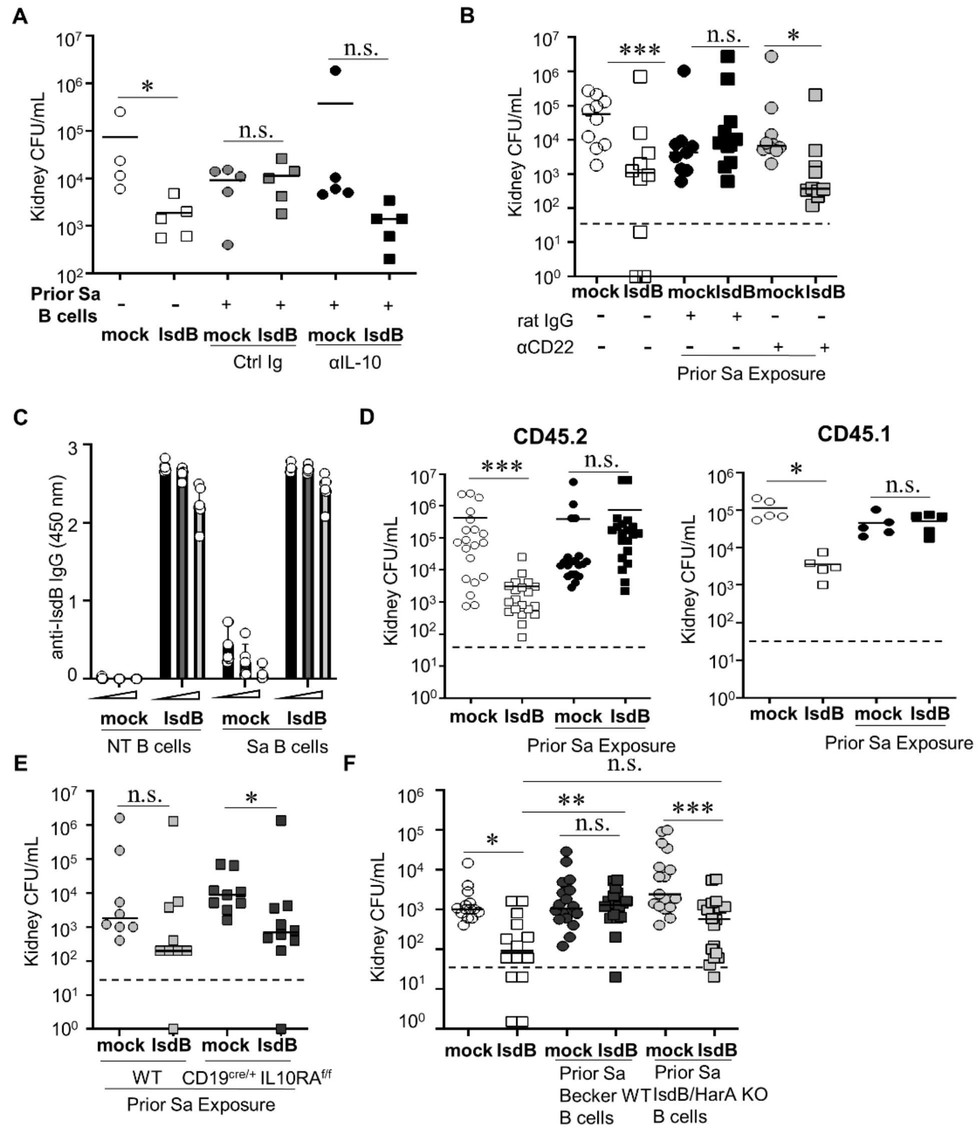

Figure S2 Sa-induced B10 cells confers vaccine interference

(A) IL-10-neutraling antibody abrogates suppressive B cell effect on IsdB vaccination. Naïve mice were adoptive transferred B cells from Sa-exposed mice, then IsdB vaccinated in the presence of Ctrl IgG or αIL-10, then challenged as per Figure 1A. Kidney CFU were measured (n=4-5 per mouse group).

(B) B10 depletion in Figure 2C restored IsdB vaccine efficacy. Mice received either Ctrl IgG or αCD22 at the time of immunization. Kidney CFU post Sa challenge were measured (n=7-10 per mouse group).

(C) D22 Titer of serum IsdB antibodies in mice that were transferred B cells from Sa-infected mice (x3), then IsdB vaccinated as in Figure 2D.

(D) De novo developed B cells are non-protective in mice exposed to suppressive B cells. The experiment was performed as per Figure 2D. CD45.1 or CD45.2 B cells were isolated 7 days after the last vaccination and transferred into naïve C57BL/6 mice followed by Sa (LAC) challenge. Kidney CFU are measured.

(E) Kidney bacterial burden in WT $CD19^{cre/+}$ mice or $CD19^{cre/+}$ $IL\text{-}10RA^{f/f}$ that were infected and IsdB vaccinated as per Figure 1A (n=8-10 per mouse group).

(F) Lack of B cell suppression of IsdB vaccine when IsdB/HarA mutant Becker strain used in prior infection as in Figure 1A followed by serum adoptive transfer. WT Becker was used in final challenge and kidney CFU were measured. The graph represents median values from three independent experiments (n=15 per mouse group).

Bars represent group median; each point represents an individual mouse; dashed lines indicate the limit of detection (A to B and D to F). Bar represents group means; error bars represent means ± SD (C). n.s., not significant, $*p < 0.05$, $**p < 0.01$, and $***p < 0.001$; one-way ANOVA followed by Bonferroni multiple comparison adjustment (A to F).

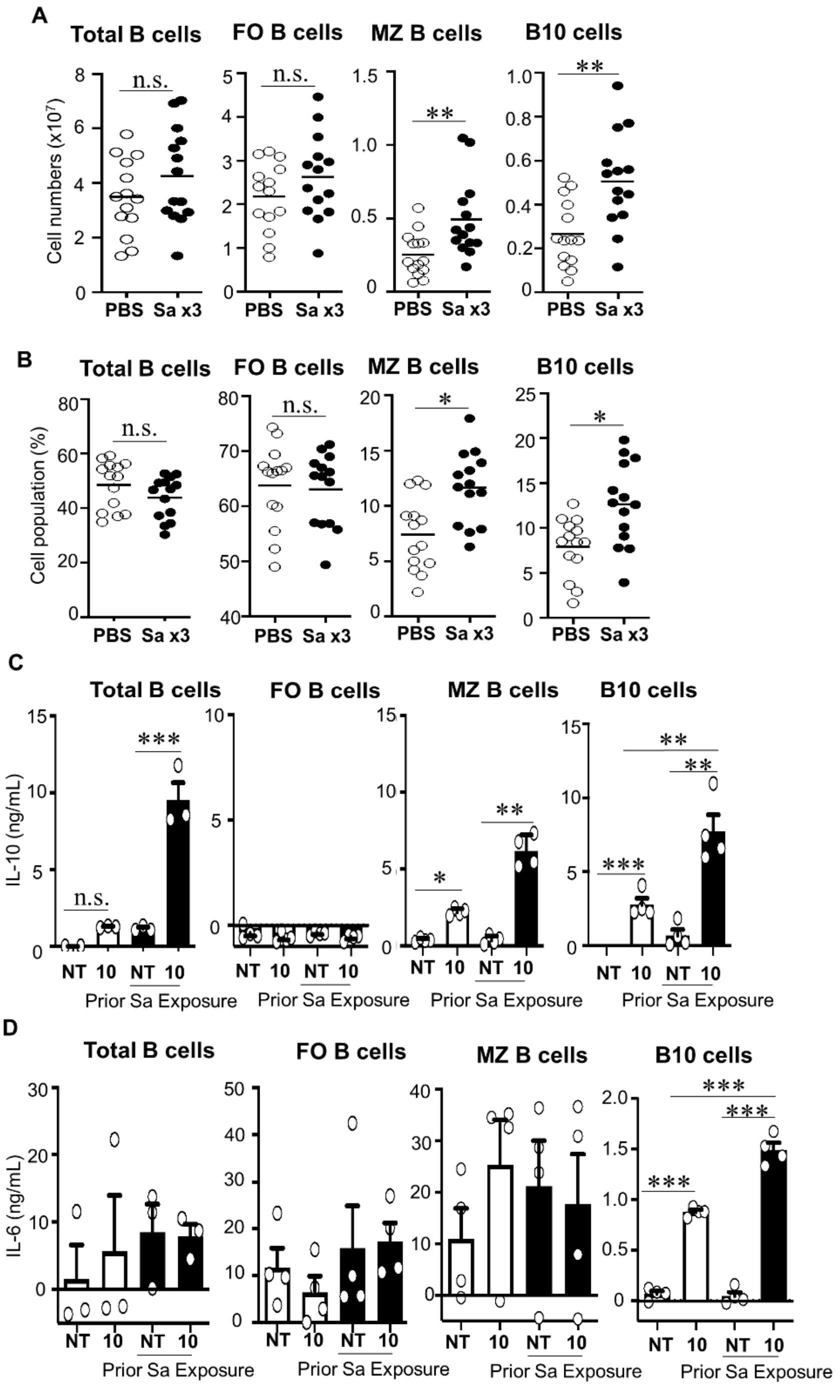

Figure S3 Phenotype of B cells in prior Sa-infected mice

(A-B) Quantification of number (A) and percentage (B) of splenic $B220^{+}$ (total), $CD19^{+}CD2^{low}CD23^{+}$ (FO), $CD19^{+}CD21^{high}CD23^{-}$ (MZ) B cells and $CD19^{+}CD1d^{high}CD5^{+}$ (B10 cells) from control or LAC infected (Sa x3) mice (n=14 per mouse group).

(C-D) IL-10 (C) or IL-6 (D) in culture supernatants of splenic B10 cells isolated from naïve or Sa-infected mice, and then treated for 16 hr with heat-killed LAC at MOI:10 or buffer control.

Bars represent group median; each point represents an individual mouse (A-B). Bar represents group means; error bars represent means ± SD (C and D). n.s., not significant, $*p < 0.05$, $**p < 0.01$, and $***p < 0.001$; Student's t test (A to B) or one-way ANOVA followed by Bonferroni multiple comparison adjustment (C to D).

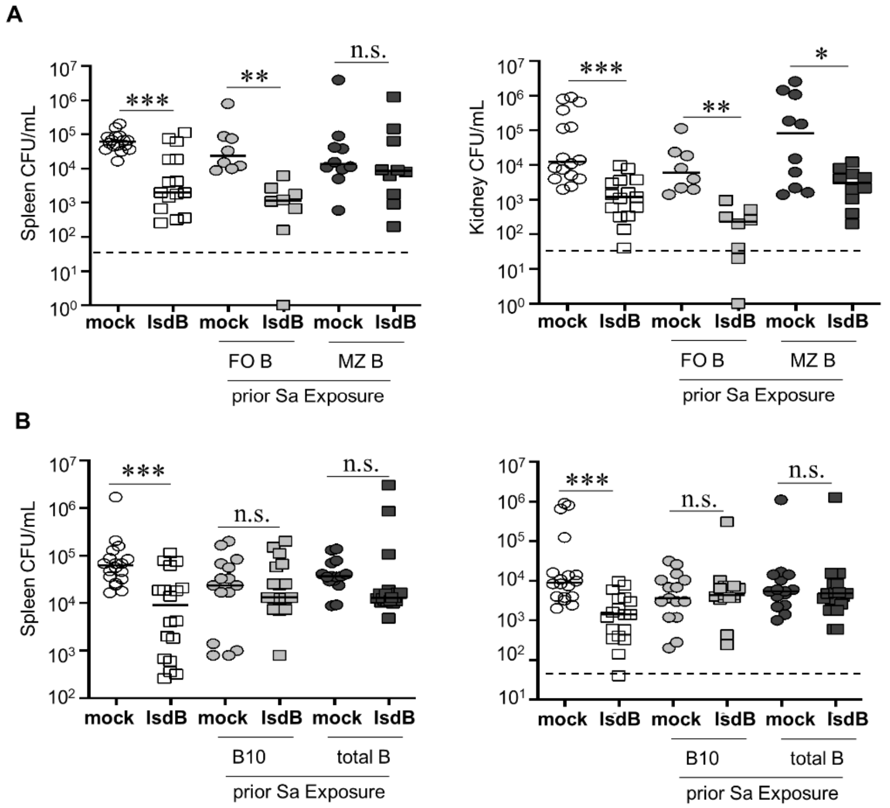

Figure S4 Adoptive transfer of Sa-experienced B cells confers vaccine suppression

(A) MZ but not FO B cells suppressed IsdB vaccination. Naïve mice were injected with $2x10^6$ FO or MZ B cells from Sa-infected mice, and then IsdB immunized and challenged with LAC. Tissue CFU were measured. The graph represents median values from two independent experiments (n=10 per mouse group from three independent experiments).

(B) B10 cells suppress IsdB vaccination. Naïve mice were injected with $5x10^5$ B10 cells or $2x10^7$ total B cells, and then IsdB immunized and challenged with LAC. Tissue CFU were measured. The graph represents median values (n-15 per mouse group from three independent experiments).

Bars represent group median; each point represents an individual mouse; dashed lines indicate the limit of detection (A-B). n.s., not significant, $*p < 0.05$, $**p < 0.01$, and $***p < 0.001$; one-way ANOVA followed by Bonferroni multiple comparison adjustment (A and B).

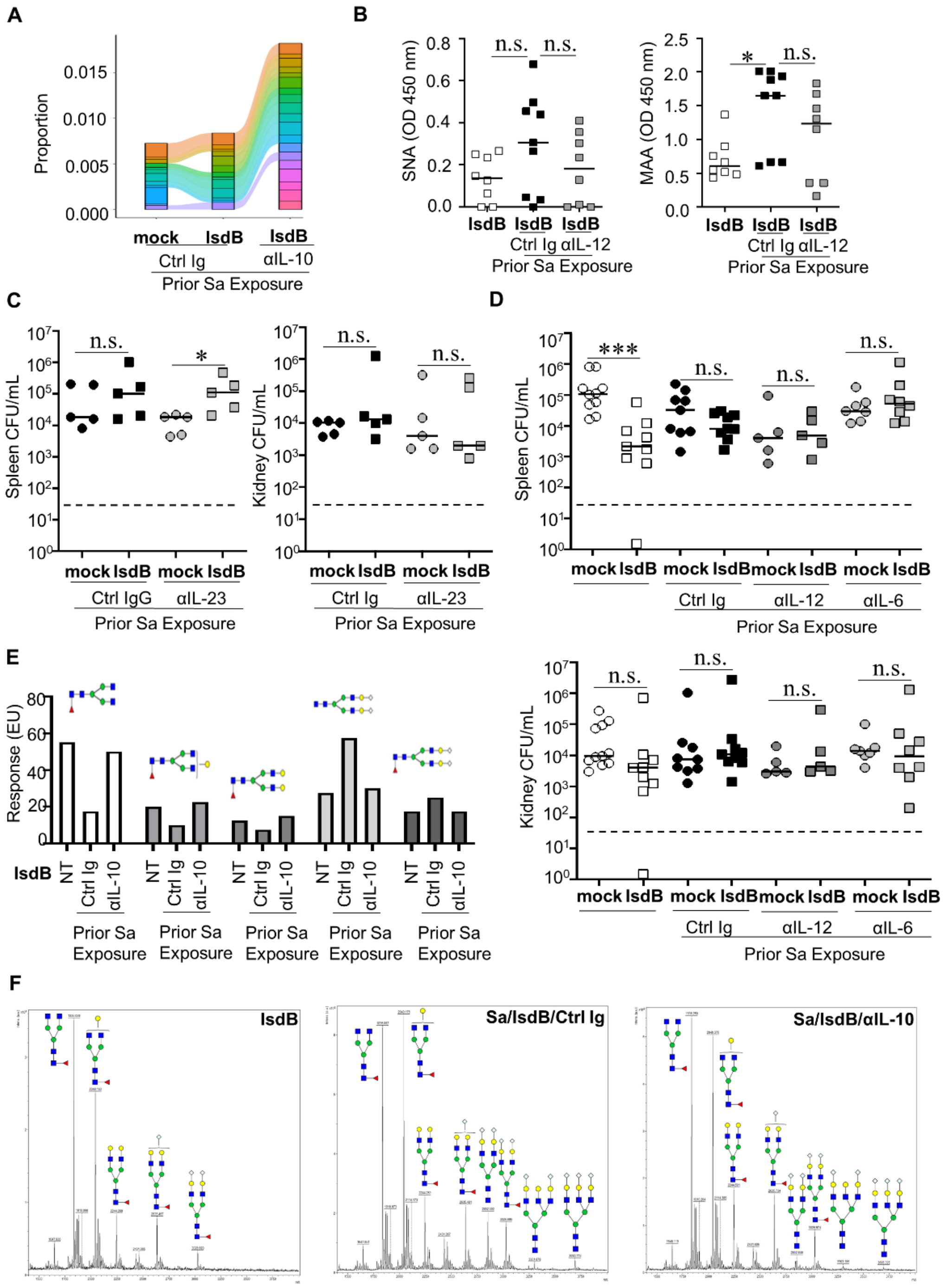

Figure S5 N-glycan analysis of anti-Sa antibodies

(A) Alluvial plot showing the top 30 clonotypes from Sa/mock, Sa/IsdB, and Sa/IsdB/αIL-10. Experiment was performed as in Figure 1D. After the final Sa challenge, splenic B cells were isolated for single cell sequencing and analysis of clonotypes based on CDR3 sequences.

(B) Effect of IL-12 neutralizing antibody on IsdB antibody sialylation. Serum IsdB antibodies from Ctrl IgG or αIL-12 antibody-treated, Sa/IsdB vaccinated mice were assessed for α-2,6 or α-2,3 sialylation by SNA and MAA lectin ELISA.

(C) Effect of IL-23 neutralizing antibody on IsdB vaccine efficacy in Sa-exposed mice performed as per Figure 1D in the presence of αIL-23 or control IgG (Ctrl Ig) during immunization.

(D) Effect of IL-12 or IL-6 neutralizing antibody on IsdB vaccine efficacy in Sa-exposed mice performed as per Figure 1D in the presence of αIL-12, αIL-6, or control IgG (Ctrl Ig) during immunization.

(E) Graphs show the level of tagged N-glycan by UPLC-FL as in Figure 3D.

(F) Mass spectrometry analysis of released N-glycan of purified IsdB antibody from IsdB mice or Sa/IsdB mice treated with Ctrl IgG or IL-10-neutralizing antibody as in Figure 1D.

Glycan nomenclature: , N-Acetylglucosamine (GlcNAc); , galactose (Gal); , mannose (Man); , Sialic acid (Neu5Gc or Neu5Ac); , Fucose (Fuc); A, antennae; S, sialic acid; F, fucose. Bars represent group median; each point represents an individual mouse (B to D). Bar represents group median; each point represents an individual mouse; dashed lines indicate the limit of detection (C and D). n.s., not significant, $*p < 0.05$, $**p < 0.01$, and $***p < 0.001$; one-way ANOVA followed by Bonferroni multiple comparison adjustment (B to D).

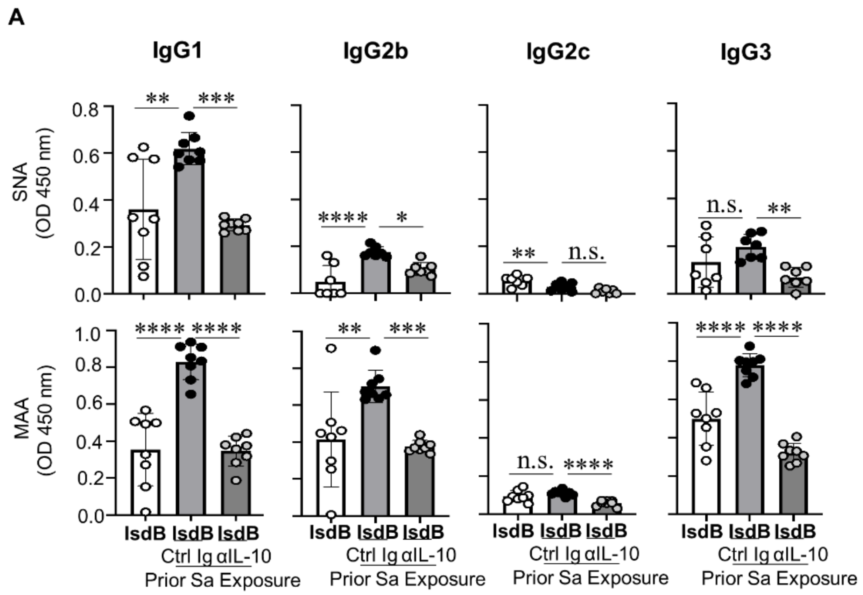

Figure S6 IL-10 increases sialylation of anti-IsdB IgG isotypes

Purified IsdB antibodies were captured on anti-IgG1, IgG2b, IgG2c and IgG3 coated plates, and the levels of sialylation were assessed by MAA and SNA lectin binding (n=8 per group).

Bars represent group median; each point represents an individual mouse. n.s., not significant, $*p < 0.05$, $**p < 0.01$, and $***p < 0.001$; one-way ANOVA followed by Bonferroni multiple comparison adjustment.

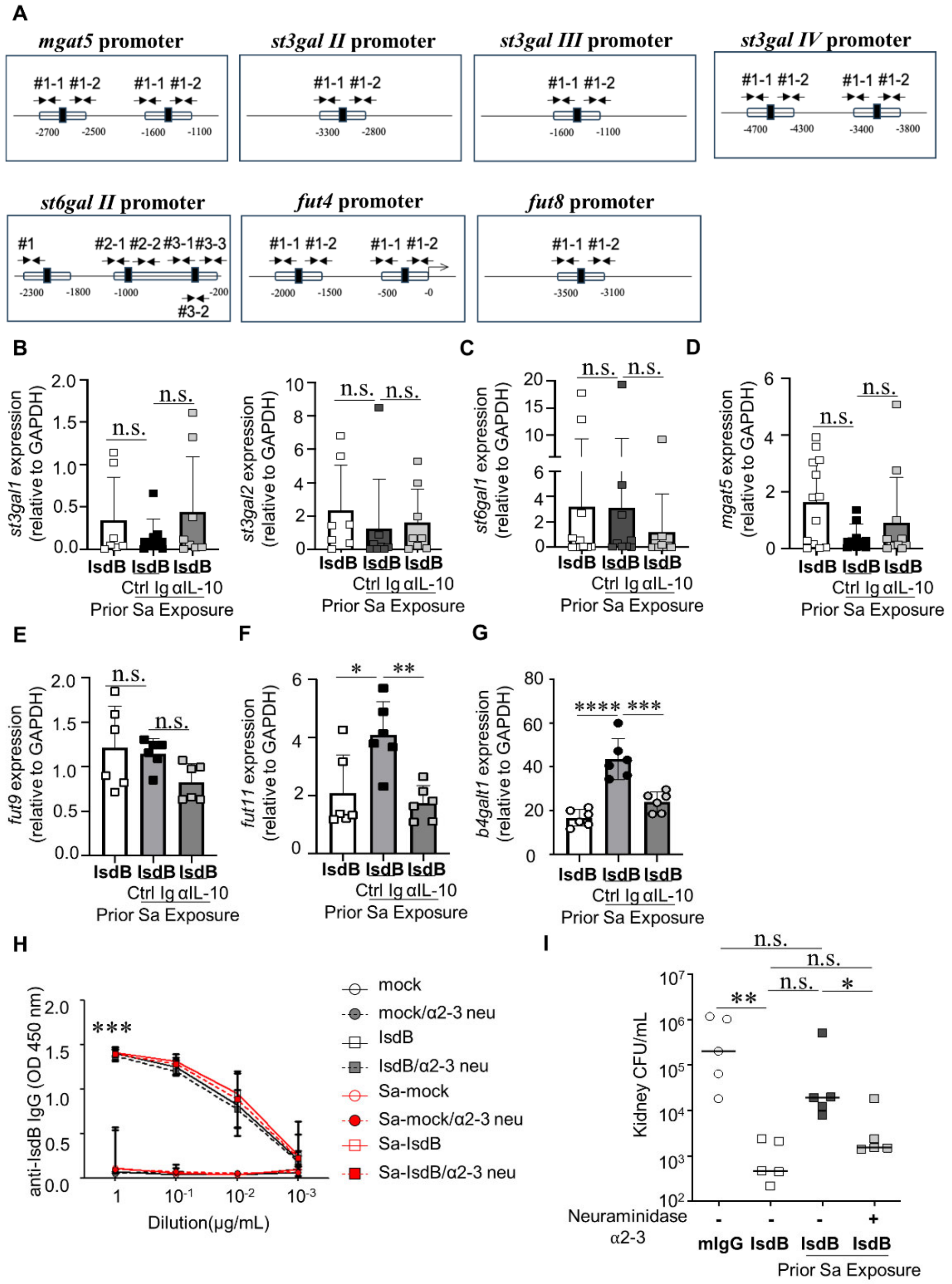

Figure S7 IL-10 effect on B cell glycosyltransferase expression and IsdB antibody function

(A) The schematics show the location of primers amplifying STAT3 binding sites on promoters of glycotransferase genes.

(B to G) Effect of αIL-10 antibody treatment on splenic B cell sialyltransferase (B-C), *Mgat5* (D), fucosyltransferase (fut) (E-F) and galactosyltransferase (galt) (G) gene expression. Experiment performed as in Figure 1D.

(H) ELISA was used to measure the rIsdB binding profile of antibodies from Figure 1A with or without α2-3 neuraminidase treatment.

(I) Effect of IsdB antibody desialylation on anti-Sa immunity in vivo. Naïve mice were injected with α2-3 neuraminidase- or control- treated, purified Sa/IsdB antibody, then infected with LAC (n=5 per mouse group). Shown are kidney CFU.

Bars represent group means; error bars represent means ± SD (B to H). Bar represents group median; each point represents an individual mouse (I). n.s., not significant, $*p < 0.05$, $**p < 0.01$, and $***p < 0.001$; one-way ANOVA followed by Bonferroni multiple comparison adjustment (B to I).

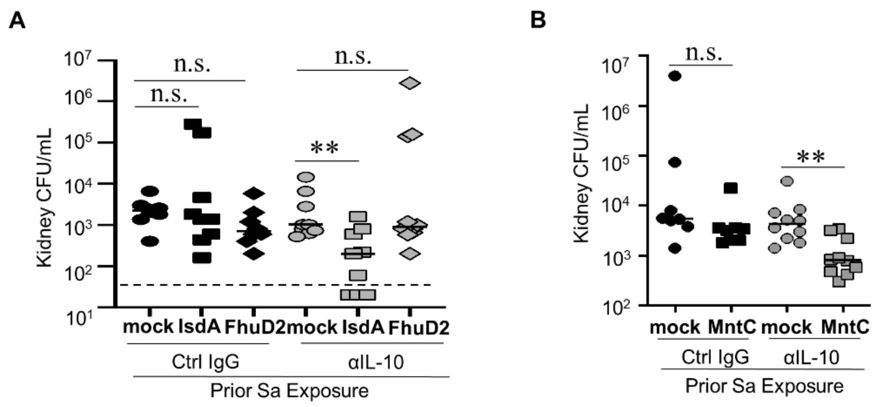

Figure S8 Neutralizing IL-10 restores anti-Sa immunity with IsdA, FhuD2 and MntC vaccines in Sa-infected mice

(A) Effect of αIL-10 antibody treatment on anti-Sa immunity conferred by IsdA or FhuD2 vaccination in Sa-exposed mice (n=7-10 per mouse group) as per Figure 5A.

(B) Effect of αIL-10 antibody treatment on anti-Sa immunity in kidneys conferred by MntC vaccination in Sa-exposed mice (n=7-10 per mouse group). as per Figure 5B.

Bars represent group median; each point represents an individual mouse; dashed lines indicate the limit of detection (A and B). n.s., not significant, $*p < 0.05$, $**p < 0.01$, and $***p < 0.001$; one-way ANOVA followed by Bonferroni multiple comparison adjustment (A and B).

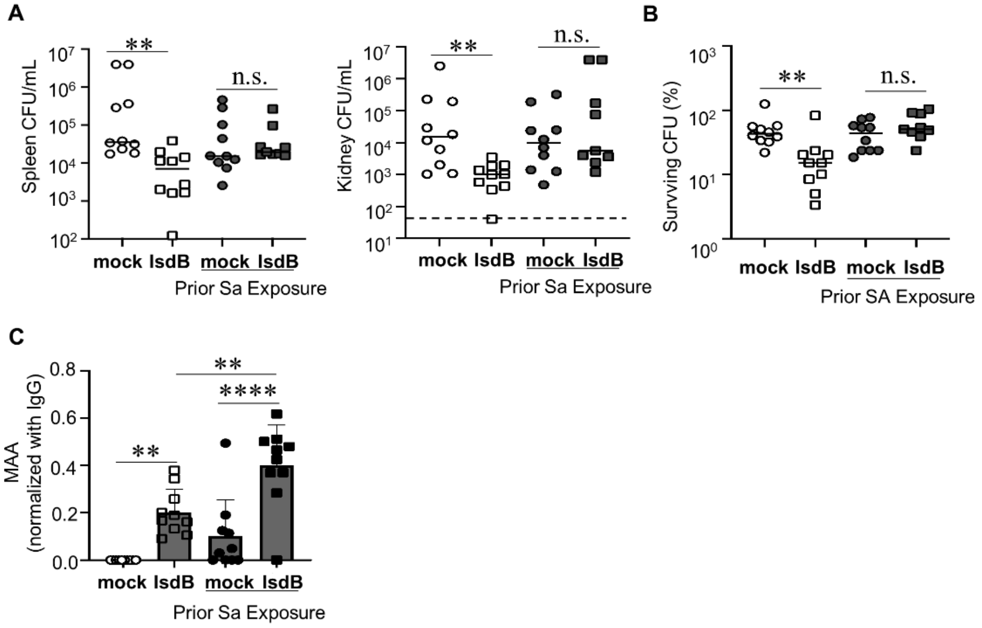

Figure S9 i.m. immunization of Sa-infected mice enhances α-2,3 sialylation on IsdB antibodies

(A) Bacterial burden 24 hr post infection in mice that have been infected 3 times with LAC, immunized i.m. with IsdB vaccine and then LAC challenged as per Figure 1A (n=9-10 per mouse group from two independent experiments).

(B) Opsonophagocytosis of Sa (LAC) by primary mouse neutrophils in the presence of sera from mice immunized in Figure S9A. The graph represents mean values ± SD from three independent experiments.

(C) Sialylation of IsdB antibodies from Figure S9A (n=9-10 per group) as assessed by MAA lectin binding normalized to IgG titer.

Bar represents group median; each point represents an individual mouse; dashed lines indicate the limit of detection (A). Bars represent group median; each point represents an individual mouse (A to C). n.s., not significant, $*p < 0.05$, $**p < 0.01$, and $***p < 0.001$; one-way ANOVA followed by Bonferroni multiple comparison adjustment (A to C).
